# Integrated metabolomic and transcriptomic profiling reveals the tissue-specific flavonoid compositions and their biosynthesis pathways in *Ziziphora bungeana*

**DOI:** 10.1186/s13020-020-00354-6

**Published:** 2020-07-16

**Authors:** Jiang He, Weijun Yang, Bo Cheng, Lina Ma, Dilinuer Tursunjiang, Zimian Ding, Yong Li, Zhaofeng Wang, Yimian Ma, Guan Li

**Affiliations:** 1 College of the Life Science and Technology, Xinjiang University, No.666, Shengli Road, Tianshan District, Urumqi, 830046 China; 2 Key Laboratory of Uygur Medicine, Xinjiang Institute of Materia Medica, No. 140, Xinhua North Road, Tianshan District, Urumqi, 830004 China; 3 Institute of Medicinal Plant Development, Chinese Academy of Medical Sciences & Peking Union Medical College, No. 151, Malianwa North Road, Haidian District, Beijing, 100193 China; 4 College of the Food Science and Pharmacy, Xinjiang Agricultural University, Saybagh District, Urumqi, 830052 China

**Keywords:** *Ziziphora bungeana*, Flavonoid, Tissue-specific, Transcriptome, Gene expression, Phylogenetic analysis, Biosynthesis

## Abstract

**Background:**

*Ziziphora bungeana* Juz. is a folk medicine from the Xinjiang Uygur Autonomous Region. The herb or the aerial parts of it have been used to medicinally treat cardiovascular diseases. Flavonoids are the main pharmacologically active ingredients in *Z*. *bungeana*. Identification of the tissue-specific distribution of flavonoids in *Z*. *bungeana* is crucial for effective and sustainable medicinal use of the plant. Furthermore, understanding of the biosynthesis pathways of these flavonoids in *Z*. *bungeana* is of great biological significance.

**Methods:**

The flavonoids from different tissues of *Z. bungeana* were identified using liquid chromatography-tandem mass spectrometry (LC–MS/MS). The full-length transcriptome of *Z. bungeana* was determined using a strategy based on a combination of Illumina and PacBio sequencing techniques. The functions of differentially expressed unigenes were predicted using bioinformatics methods and further investigated by real-time quantitative PCR and phylogenetic relationship analysis.

**Results:**

Among the 12 major flavonoid components identified from *Z. bungeana* extracts, linarin was the most abundant component. Nine flavonoids were identified as characteristic components of specific tissues. Transcriptome profiling and bioinformatic analysis revealed that 18 genes were putatively involved in flavonoid biosynthesis. The gene expression and phylogenetic analysis results indicated that *ZbPALs*, *Zb4CL3*, *ZbCHS1*, and *ZbCHI1* may be involved in the biosynthesis of the main flavonoid intermediate. *ZbFNSII*, *ZbANS*, and *ZbFLS* may be involved in the biosynthesis of flavones, anthocyanins, and flavonols, respectively. A map of the biosynthesis pathways of the 12 major flavonoids in *Z. bungeana* is proposed.

**Conclusions:**

The chemical constituent analysis revealed the compositions of 9 characteristic flavonoids in different tissues of *Z. bungeana*. Linarin can be hydrolysed into acacetin to exert a pharmaceutical role. Apigenin-7-*O*-rutinoside is hypothesised to be the precursor of linarin in *Z. bungeana*. There was greater content of linarin in the aerial parts of the plant than in the whole herb, which provides a theoretical basis for using the aerial parts of *Z. bungeana* for medicine. These results provide a valuable reference for further research on the flavonoid biosynthesis pathways of *Z. bungeana* and will be significant for the effective utilisation and ecological protection of *Z. bungeana*.

## Background

*Ziziphora bungeana* Juz. is a perennial semi-shrub. It is a subspecies of *Z. clinopodioides* Lam. and belongs to the genus *Ziziphora* L. in the Labiatae family. The plant is mainly distributed in Kazakhstan, China, Central Asia, and Mongolia, and is used to treat hypertension and heart diseases [1]. In China, it only grows in the Xinjiang Uygur Autonomous Region and has long been used as a folk medicine to treat diseases such as cardiopathy, hypertension, fever, headache, insomnia, and cardiopalmus [2, 3]. The main active constituents of *Z. bungeana* are flavonoids and essential oils [1–6]. The essential oils of *Z. bungeana* have antioxidant and antimicrobial activities [1, 4]. The flavonoids of *Z. bungeana* have significant protective functions for vascular endothelial cells [5–8].

Flavonoid represents a large subgroup of plant secondary metabolites with various biological activities. The major groups of flavonoids include flavones, flavanones, isoflavones, flavonols, chalcones, and anthocyanins. The flavone group is one of the largest groups of flavonoids. Flavones have hundreds of structures with diverse pharmacological properties. In recent years, there has been rapid development of flavone-based therapeutic agents [9, 10]. The flavonoid ingredients of *Z. bungeana*, including some flavones, have significant effects on cardiovascular diseases. Previous studies have reported that apigenin, chrysin, and ethyl 4-coumarate of *Z. bungeana* are effective vasorelaxant compounds [11]. Linarin (buddleoside) and acacetin can also protect myocardial tissue from ischemia–reperfusion injury [12–14]. Traditionally, the whole herb of *Z. bungeana* has been used as a medicine to treat cardiovascular diseases. In China, *Z. bungeana* resources are limited and have been decreasing over time. To protect the *Z. bungeana* resources, many areas now only use the aerial parts of the plant for medicine [2, 3]. However, whether there are differences in the flavonoid composition of the aerial parts relative to the whole herb remains unknown. Recently, studies have reported that among the three active ingredients of *Z. bungeana* (caffeic acid, rosmarinic acid, and linarin), linarin was the most abundant active ingredient [15], with the highest contents of linarin observed in the inflorescence [16]; however, it is still not clear whether linarin is the major flavonoid constituent in *Z. bungeana*. Aside from linarin, understanding of the tissue-specific distribution of other flavonoids is lacking. Therefore, it is necessary to comprehensively study the major flavonoids in different tissues of *Z. bungeana*.

Over the past decade, studies have revealed the core biosynthesis pathways of flavonoids in various plants such as *Arabidopsis* [17], rice [17], lettuce [18], *Camellia sinensis* [19], *Salvia miltiorrhiza* [20], *Oroxylum indicum* [21], *Chrysanthemum morifolium* [22], *Chrysanthemum indicum,* and *Scutellaria baicalensis* [23, 24]. The biosynthesis pathways of *Chrysanthemum indicum*’*s* bioactive ingredients, including apigenin, luteolin, and linarin, have been elucidated. Linarin is thought to be biosynthesised from acacetin-7-*O*-glucoside [23]. However, it is still not known whether linarin in *Z. bungeana* is biosynthesised from acacetin-7-*O*-glucoside. In this study, metabolomic and transcriptomic profiling was used to identify the tissue-specific flavonoids in *Z. bungeana* and their biosynthesis pathways. A combination of Illumina- and SMRT-based sequencing techniques was used to identify the full-length unigenes [25–28]. Real-time quantitative PCR and phylogenetic relationship analyses were used to determine the functions of flavonoid biosynthesis genes in *Z. bungeana*. Our analysis revealed that linarin is the major flavonoid in *Z. bungeana* and it is most probably biosynthesised from apigenin-7-*O*-rutinoside. A map of the biosynthesis pathways of the major flavonoid ingredients, and the involved genes, is proposed.

## Materials and methods

### Collection of plant materials

The plant materials were three-year-old *Z. bungeana* plants grown in the Xinjiang Institute of Materia Medica; all were of similar growth status. The plants were cultivated in plastic baskets (35 × 45 × 35 cm) with soil from the natural habitat of *Z. bungeana*. The root, stem, leaf, and inflorescence of *Z. bungeana* were collected on July 2, 2016. The infructescence was collected on July 30, 2016. Each tissue sample is with three biological replicates. All tissues were cut into small pieces, placed into liquid nitrogen for an hour, and then stored in an ultra-low temperature freezer (− 80 °C) before high throughput sequencing. Meanwhile, part of each sample was dried at 60 °C for 48 h, crushed, and sifted passing a 40 mesh sieve. Each powder (approximately 1 g) was extracted twice by reflux extraction in 50 ml 70% ethanol (v/v) for 90 min. After filtering, the extracts were mixed and diluted to 100 ml for further LC–MS/MS analysis.

### LC–MS/MS analysis

Caffeic acid, hyperoside, rutin, rosmarinic acid, quercetin, apigenin, chrysin, and genkwanin standards were purchased from the National Institutes for Food and Drug Control (Beijing, China). Linarin, luteolin, and kaempferol were purchased from ChromaDex (Irvine, United States). Acacetin was purchased from Spring & Autumn Biological Engineering (Nanjing, China). High-performance liquid chromatography (HPLC) was performed using an Agilent 1260 HPLC system. The separation and purification for total flavonoids of *Z. bungeana* was following our reported method [15, 16, 29]. For each sample, 2 ml of extracted solution was filtered through a 0.22 μm membrane and injected into the HPLC system. The separation was performed on a 150 × 4.6 mm extend C18 column held at 30 °C; the flow rate was 0.8 ml/min. The optimal mobile phase consisted of A (CH3COOH/H2O, 0.2:100, v/v) and B (CH3OH). The optimized HPLC gradient elution conditions were as follows: 0–8 min, 5% B; 8–60 min, 5–80% B; 60–65 min, 80–95% B; 65–70 min, 95–5% B; 70–80 min, 5% B. The injection volume was 15 μl and the detection wavelength was 340 nm. Flavonoids were quantified by calculating the area of each peak and comparing them with standard curves obtained from pure compounds.

HPLC–MS/MS was performed using an LC/MSD-Trap-SL electrospray ion trap LC/MS (1100 Series LC/MSD Trap, a complete LC–MS/MS). All HPLC system devices were from the Agilent 1100 series and consisted of a vacuum degasser (model G1322A), a quaternary pump (model G1311A), an autosampler (model G1329A), a thermostated column compartment (model G1316A), and a diode array detector (model G1315A). The settings were as follows: ion source type, APCI (positive mode); nebulizer pressure, 40 psi; dry gas temperature, 350 °C; dry gas flow, 9.0 l/min; APCI Vap temperature, n/a; corona current, n/a; capillary voltage, 3500 V. The LC/MSD-Trap software 5.3 was used for data analysis. The relevant literature on *Z. bungeana* published in CNKI, PubMed, SciFinder, and ChemSpider was used to establish the database. The chemical structures were further confirmed based on fragment ions [30–34].

### Illumina-based RNA sequencing

Total RNA was extracted from the root (Rf), stem (Sf), leaf (Lf), inflorescence (Ff), and infructescence (Faf) of *Z. bungeana* using an RNAprep pure plant kit (Tiangen, China). The RNA from each part of the plant was used to isolate mRNAs and construct 15 cDNA libraries (Rf1, Rf2, Rf3, Sf1, Sf2, Sf3, Lf1, Lf2, Lf3, Ff1, Ff2, Ff3, Faf1, Faf2, and Faf3). The mRNA isolation kit used was the TruSeq Stranded mRNA LT Sample Prep Kit (Illumina, USA). Then, the mRNAs were broken into 200–300 bp fragments using the ion-interruption method and were reverse-transcribed into double-strand cDNAs with random hexamer primers. After end repair, adaptor ligation, and addition of index codes for each sample, the cDNAs with sizes between 300 and 400 bp were amplified and enriched as a cDNA library. The purity and quality of the 15 cDNA libraries were measured using an Agilent 2100 Bioanalyser. Afterward, the 15 cDNA libraries were sequenced using the Illumina NextSeq 500 platform. As a result, each sample generated about 4G of raw data. The raw sequence data were deposited in the SRA database with accession numbers as follows: Rf1, SRR10352318; Rf2, SRR10352311; Rf3, SRR10352310; Sf1, SRR10352309; Sf2, SRR10352308; Sf3, SRR10352307; Lf1, SRR10352306; Lf2, SRR10352305; Lf3, SRR10352304; Ff1, SRR10352317; Ff2, SRR10352316; Ff3, SRR10352315; Faf1, SRR10352314; Faf2, SRR10352313; Faf3, SRR10352312.

### SMRT-based RNA sequencing

For Pacbio-based RNA sequencing (RNA-seq), the samples for Pac-Bio library construction were the mixed RNAs from all five plant samples. The full-length cDNAs were synthesised using a SMARTer PCR cDNA Synthesis Kit (Clontech, USA). The cDNA library was constructed using the SMRTbell™ Template Prep Kit 1.0, following the manufacturer’s instructions. The cDNA library was sequenced using a Sequel™ Sequencing Kit 2.0 with a PacBio Sequel system (Pacific Biosciences, USA). The raw sequence data were deposited in the SRA database and are available under accession number SRR10352319.

### Sequence assembly and unigene annotation

After filtering the adaptors and low-quality reads, the clean reads generated by the PacBio Sequel system were assembled and clustered by sequence recognition and isoform-level clustering using CD-HIT software; this was used as the reference transcriptome [35]. Then, the clean reads generated by the Illumina sequencing platform were mapped to the reference transcriptome; the unmapped sequences were assembled using Trinity software [27]. Lastly, the unigenes acquired by the PacBio Sequel system and the Illumina sequencing platform were further combined and clustered using CD-HIT software to generate assembled unigenes. The unigenes were annotated based on their sequence similarity to other genes or proteins in public databases such as NCBI Non-Redundant Protein (NR), Gene Ontology (GO), Kyoto Encyclopedia of Genes and Genomics (KEGG), Evolutionary Genealogy of Genes: Non-supervised Orthologous Groups (eggNOG), and Swiss-Prot.

### Analysis of differentially expressed unigenes

The clean reads acquired by the Illumina platform were mapped to the assembled transcript sequences using the software RSEM [36]. The number of reads mapped to each gene was counted and normalised into an FPKM (Fragments Per Kilobase of transcript per Million fragments mapped) value using Cufflinks [37]. The unigene expression levels were evaluated based on the FPKM values. The differentially expressed genes (DEGs) between every two samples were determined using the software DEseq [38]. Significant expression differences between different samples were determined based on a |log2 (fold change)| > 1 and a significance p value < 0.05. Volcano plots and a heatmap were constructed using the ggplots2 software package and the pheatmap package of R.

### Identification of genes involved in flavonoid biosynthesis

We classified the DEGs based on GO categories and KEGG pathway analysis. The DEGs enriched in the GO categories and KEGG pathways related to flavonoid metabolism were selected and analysed. We searched for unigenes involved in flavonoid biosynthesis via their sequence similarity with flavonoid biosynthesis enzymes in other plant species using the tBLASTn algorithm (E < 1e−5). These unigenes were further screened based on their ORF (open reading frame) lengths and their expression levels (FPKM values). The screening criteria are: 1. ORF > 300 bp; 2. FPKM value > 10. Primers were designed for amplifying the coding sequences of 18 unigenes that satisfied the two criteria (Additional file 1: Table S1). Then, the amplified sequences that agreed with the predictions were submitted to the Genbank group for an arrangement of accession numbers.

### Sequence feature and phylogenetic relationship analysis

The coding sequences of genes and predicted proteins were acquired using the software Vector NTI 11.5 [39]. The phylogenetic tree was constructed using the neighbour-joining clustering method with 1000 bootstrapped replications [40]. The phylogenetic tree was customised and annotated using the online tool Evolview v2 [41].

### Real-time quantitative PCR

Total RNA was extracted from *Z. bungeana* tissues from the root (Rf), stem (Sf), leaf (Lf), and inflorescence (Ff) using an RNAprep Pure Plant kit for polysaccharides and polyphenolics-rich materials (TIANGEN, Beijing, China). RNA integrity was analysed on 1% agarose gel and RNA quantity was determined using a NanoDrop 2000c spectrophotometer (Thermo Scientific, USA). Reverse transcription was carried out with PrimeScript™ RT Master Mix (TAKARA, Dalian, China). Quantitative real-time PCR (qPCR) was carried out in triplicate reactions using a BIO-RAD CFX96 system. The qPCR experiment used the TB Green^®^ Premix Ex Taq™ (Tli RNaseH Plus) reagent (TAKARA, Dalian, China) with the internal reference *ZbEF1γ* [42]. The online primer design tool Primer3Plus (http://www.primer3plus.com/cgi-bin/dev/primer3plus.cgi) was used to design primers for quantitative real-time RT-PCR. For gene family members, the primers with the best specificity were chosen (Additional file 1: Table S2).

## Results

### Identification of characteristic flavonoid components in various tissues of *Ziziphora bungeana*

**Fig. 1:**
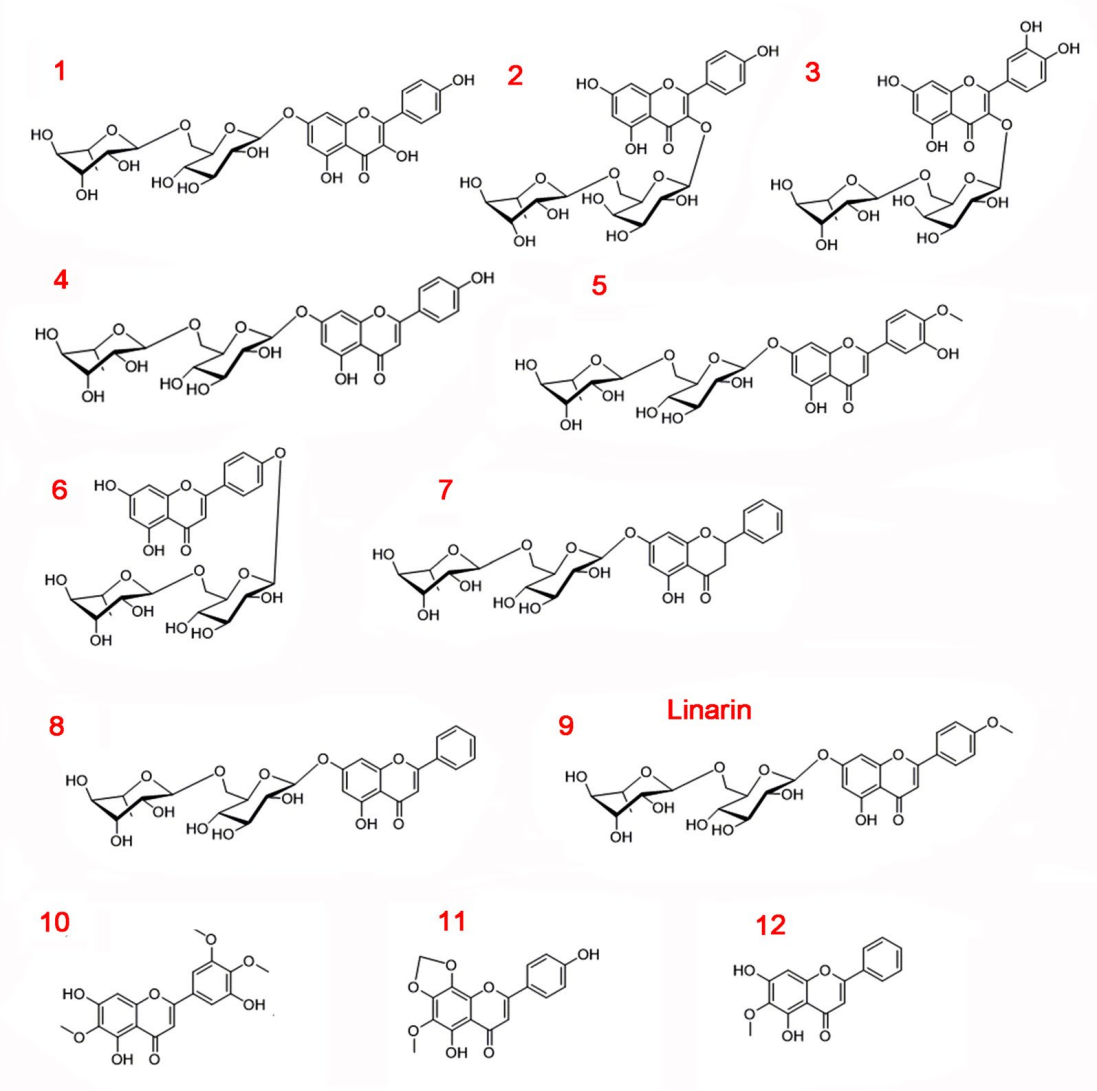
The chemical structures of flavonoid compounds identified by LC–MS/MS. The name of the 12 flavonoids (1–12) are 1: Kaempferol-7-*O*-rutinoside; 2: Kaempferol-3-*O*-rutinoside; 3: Rutin; 4: Apigenin-7-*O*-rutinoside; 5: 3ʹ-hydroxyacacetin-7-*O*-rutinoside; 6: Acacetin-4ʹ-*O*-rutinoside; 7: Pinocembrin-7-*O*-rutinoside; 8: Chrysin-7-*O*-rutinoside; 9: Linarin; 10: 5,7,3ʹ-trihydroxy-6,4ʹ,5ʹ-trimethoxyflavone; 11: 5,4ʹ-dihydroxy-6-methoxy-7,8-methylenedioxyflavone; 12: 5,7-dihydroxy-6-methoxyflavone

**Fig. 2:**
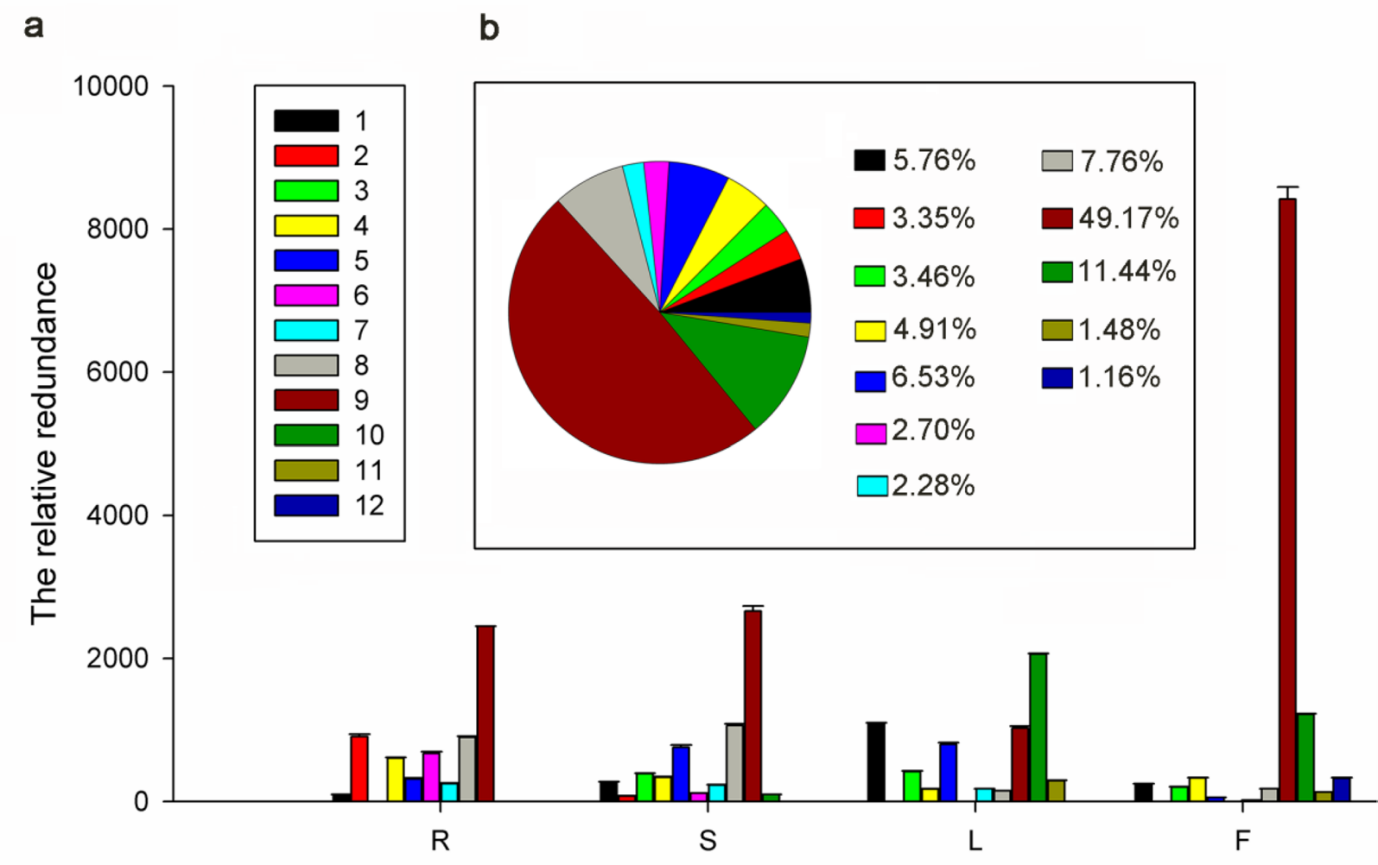
The tissue-specific distribution of the 12 flavonoid compositions from *Z. bungeana* extracts and their proportions. The legend 1–12 represent the 12 major flavonoids shown in Fig. 1. **a** The relative abundance of the flavonoids from the root (R), stem (S), leaf (L), and inflorescence (F). The number in the y-axis of the bar chart represents the relative abundance of the flavonoids calculated based on the integrated LC–MS/MS peak area. SEs (Standard Errors) were calculated from three biological replicates. **b** The relative abundance of R, S, L, and F was added together to acquire the total relative abundance values for each of the 12 flavonoids. For the 12 major flavonoids, the proportion of each flavonoid was shown as the pie chart

In total, we detected 39 chemical structures in the 70% ethanol extracts from different tissues of *Z. bungeana* plants at their fluorescence stage by LC–MS/MS (Additional file 1: Table S3). The MS data and the identification results for the 39 chemicals included 25 flavonoids and 22 possible new chemicals (the possible new chemicals are indicated by * in Additional file 1: Table S3) [5, 11, 43–55]. The total ion chromatograms for compounds in different tissues of *Z. bungeana* were shown as Figure S1 in Additional file 2. Among the chemical compounds, 12 flavonoid constituents had relatively higher contents than the others, and thus, were regarded as the major flavonoid components (Fig. 1). The 12 major flavonoid constituents were classified as flavonol glycosides (1, 2, and 3), flavone glycosides (4, 5, 6, 8, and 9), dihydroflavanone glycosides (7), and polyhydroxyflavones (10, 11 and 12). Among them, 9 (1–9) were *O*-rutinose glycosylated-type flavonoids and 6 (1, 4, 5, 7, 8 and 9) were 7-*O*-rutinoside-type flavonoids. In these flavonoids, acacetin-4′-*O*-rutinoside (6) and 5,4′-dihydroxy-6-methoxy-7,8-methylenedioxyflavone (11) are possible new compounds. The relative abundances of the flavonoids of *Z. bungeana* were analysed based on the corresponding peak areas obtained with LC–MS/MS. Based on this analysis, flavones were the major components of the 70% ethanol extracts. Of the total relative abundance of the major 12 flavonoids (1–12), linarin (9) accounted for 49.17%, 5,7,3ʹ-trihydroxy-6,4ʹ,5ʹ-trimethoxyflavone (10) accounted for 11.44%, and the other compounds accounted for no more than 10% (Fig. 2b). We also calculated the relative abundance of these compounds in the root, stem, leaf, and inflorescence of *Z. bungeana*. As shown in Fig. 2a, the relative abundance of linarin (9) was highest in the inflorescence, which indicates that the inflorescence is the primary location of linarin biosynthesis. Among the two kaempferol glycosides, kaempferol-7-*O*-rutinoside (1) was most abundant in the leaf, but kaempferol 3-*O*-rutinoside (2) was most abundant in the root, which suggests tissue-specific modification of kaempferol.

Further, relative peak area analysis revealed significant variation in the distribution of components in various tissues of *Z. bungeana* (Fig. 2a, Additional file 1: Table S4). The chemicals identified as characteristic components of specific tissues were those that had a significantly greater relative peak area (p < 0.01) together with more than 20% higher content relative to other tissues. The results indicated that linarin (9) and 5,7-dihydroxy-6-methoxyflavone (12) were the characteristic components of inflorescence; kaempferol-7-*O*-rutinoside (1), 5,7,3ʹ-trihydroxy-6.4ʹ,5ʹ-trimethoxyflavone (10), and 5,4ʹ-dihydroxy-6-methoxy-7,8-methylenedioxyflavanone (11) were the characteristic components of leaves; kaempferol 3-*O*-rutinoside (2), apigenin-7-*O*-rutinoside (4), and acacetin-4ʹ-*O*-rutinoside (6) were the characteristic components of roots; and chrysin-7-*O*-rutinoside (8) was the characteristic component of stems.

### Unigene characterisation and functional annotation

**Fig. 3:**
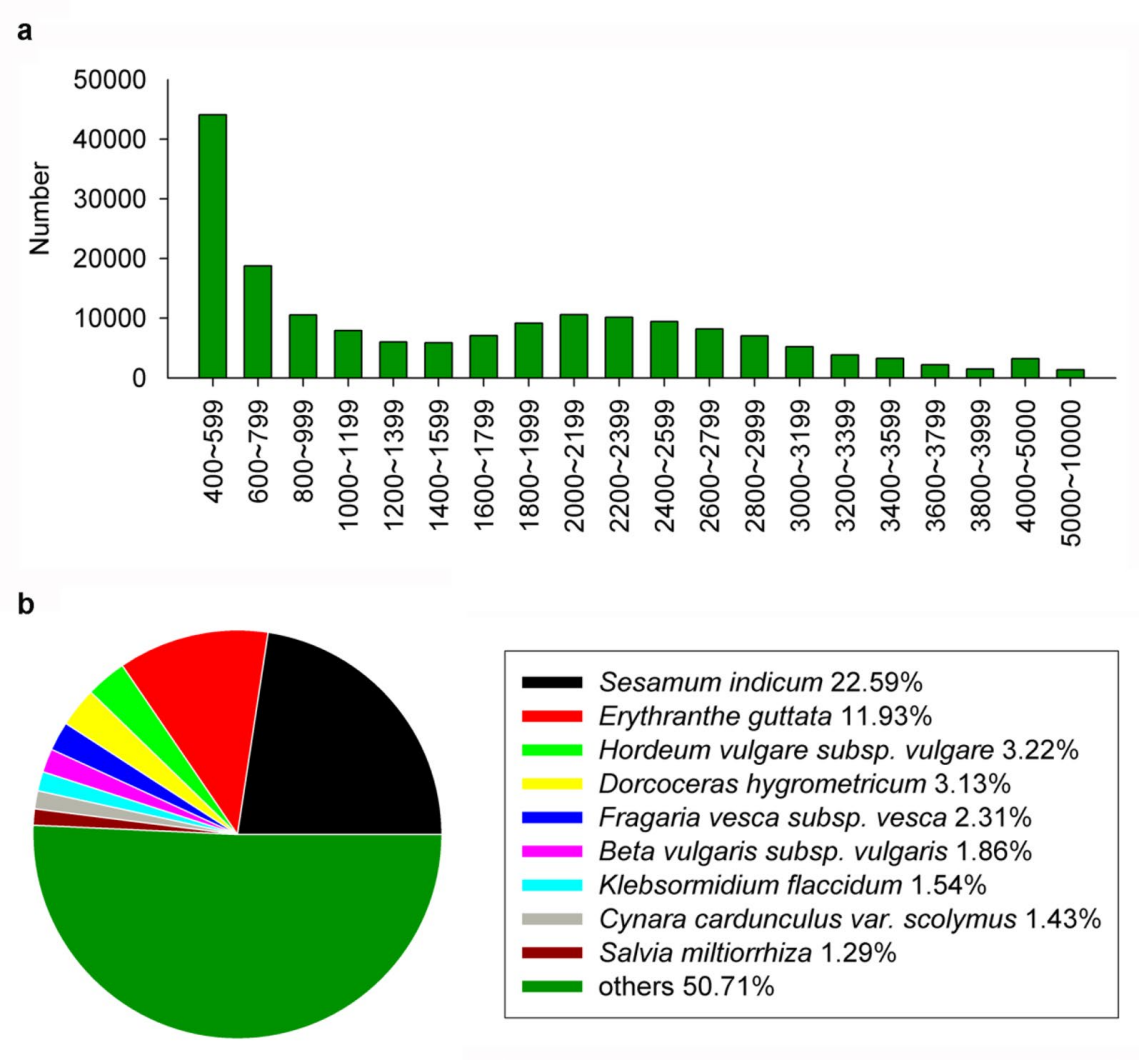
Sequence length distribution and the distribution ratio of species with top hits. **a** The number of unigenes with length between 400 and 10,000 bp. **b** The distribution ratio of the species with Blastx top hits against the Nr database

Using the Illumina- and SMRT-based high-throughput sequencing platforms, we acquired the full-length transcriptome of *Z. bungeana*, with a total of 397,182 assembled unigenes. These unigenes had an average length of 870 bp (Additional file 1: Table S5). The number distributions of these unigenes in each 200 bp range were shown as Fig. 3a. All the 397,182 unigenes were used to search against the NCBI non-redundant protein sequences (Nr), Gene Ontology (GO), Kyoto Encyclopedia of Genes and Genomics (KEGG), Evolutionary Genealogy of Genes: Non-supervised Orthologous Groups (eggNOG), and Swiss-Prot databases, and were annotated based on sequence similarities. The annotation results are shown in Additional file 1: Table S6. The Nr annotation results showed that about 225,633 unigenes had significant similarities with other genes in Genbank, accounting for 56.81% of the total number of unigenes. As shown in Fig. 3b, BlastX against the Nr database indicated that the unigenes matched many species. The top nine hits were all plant species. Although *Salvia miltiorrhiza* and *Z. bungeana* are in the same family (Labiatae), *S*. *miltiorrhiza* was only ranked 9th among the top hit species.

The levels of gene expression among the various tissues were compared. The expression levels of the unigenes were determined based on the normalised FPKM values; 42,875 DEGs were identified. The FPKM values of these DEGs were used to construct a heatmap (Additional file 2: Figure S2). The distribution and expression fold changes in these DEGs are shown as four volcano plots (Additional file 2: Figure S3). We enriched the DEGs into the GO categories and the KEGG pathways (Additional file 3). Among the DEGs between different tissues, 7757 DEGs (Rf vs. Ff), 5132 DEGs (stem vs. inflorescence, Sf vs. Ff), 6975 DEGs (leaf vs. inflorescence, Lf vs. Ff), and 7687 DEGs (infructescence vs. inflorescence, Faf vs. Ff) were categorised by their GO terms. When comparing the enriched GO terms, we found that the 10 most redundant terms were similar. Thus, we counted the numbers of DEGs in the 10 most redundant GO terms. The enriched GO terms and the number of enriched DEGs are shown in Additional file 2: Figure S4. We used the DEGs to search the KEGG pathway database and arranged the pathways. Furthermore, the significantly enriched pathways were obtained by hypergeometric distribution computation (the total unigenes were used as the reference genome). Then, the dot plots were drawn to show the 20 most significantly enriched pathways for each comparison (Additional file 2: Figures S5–S8). Among the 20 most significantly enriched KEGG pathways for Rf vs. Ff, ‘Flavonoid biosynthesis’ was ranked 13th and contained 8 DEGs. For Sf vs. Ff, ‘Flavonoid biosynthesis’ was also ranked 13th among the 20 most enriched KEGG pathways, but it contained only 6 DEGs.

### Analysis of genes involved in flavonoid biosynthesis

**Fig. 4:**
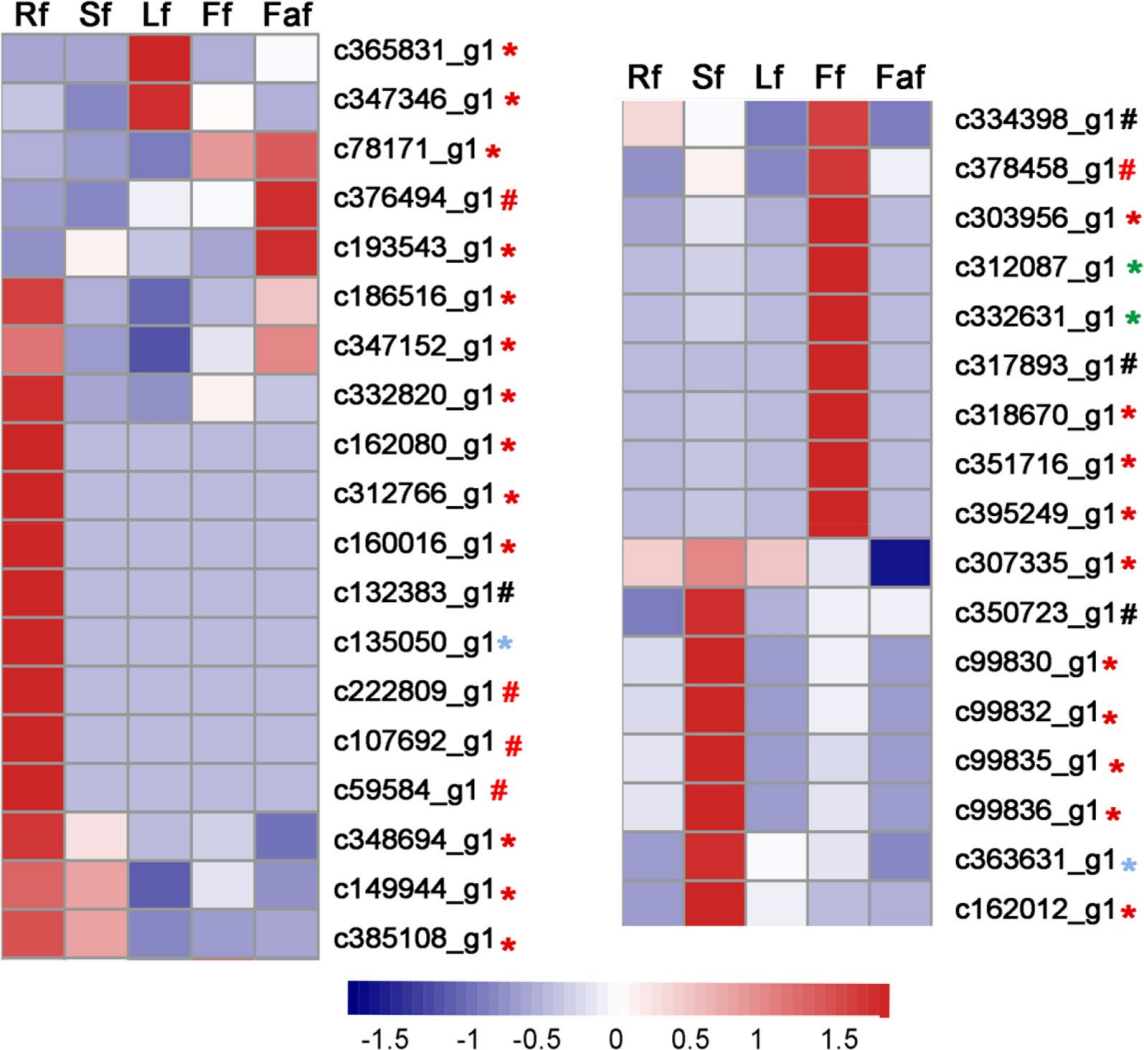
Heatmap depicting the tissue-specific expression patterns of DEGs probably involved in flavonoid biosynthesis. The heatmap was conducted from the standardized FPKM profiles of the unigenes. Rf, Sf, Lf, Ff, and Faf represent root, stem, leaf, inflorescence, and infructescence, respectively. The unigene marked using black ‘**#**’ represent DEG encoding CHS. The unigene marked using red ‘#’ represent DEG encoding CHI. The unigene marked using blue ‘*’ represent DEG encoding FLS. The unigene marked using green ‘*’ represent DEG that encode ANS. The unigene marked using red ‘*’ represent DEG that encode GT

The search of the KEGG pathways and GO categories with enriched DEGs revealed 14 DEGs enriched in the KEGG pathways related to flavonoid biosynthesis (‘ko00941 Flavonoid biosynthesis’ and ‘ko00944 Flavone and flavonol biosynthesis’) and 199 DEGs enriched in the GO categories associated with flavonoid biosynthesis (‘GO: 0009813 flavonoid biosynthetic process’ and ‘GO: 0051552 flavone metabolic process’) (Additional file 4). In these DEGs, 4 DEGs encoded chalcone synthase (CHS), 5 DEGs encoded chalcone isomerase (CHI), 2 DEGs encoded flavonol synthase (FLS), and 2 DEGs encoded anthocyanidin synthase (ANS), all of these DEGs with ORFs more than 300 bp; 23 DEGs encoded glycosyltransferase (GT) with ORFs more than 600 bp. The expression patterns of these DEGs in various tissues were analysed based on their FPKM values. As shown in Additional file 5, the unigene c334398_g1 encoding CHS had the highest FPKM value in the inflorescence. The two unigenes that encoded ANS (c312087_g1 and c332631_g1) also had high FPKM values. For these DEGs, a heatmap was constructed to show the classification of tissue-specific expression. The result indicated that 10 unigenes had inflorescence-specific expression (Fig. 4). To further analyse and identify the candidate genes that are putatively involved in flavonoid biosynthesis in *Z. bungeana*, the key enzymes, such as phenylalanine ammonia-lyase (PAL), cinnamate 4-hydroxylase (C4H), 4-coumarate-CoA ligase (4CL), and flavone synthase II (FNSII), from *Arabidopsis* and *Salvia miltiorrh*iza were used to search for homologous unigenes in the assembled *Z. bungeana* unigene set using the tBLASTn algorithm (E < 1e−5). The results revealed 18 candidate genes with ORFs > 300 bp and FPKM values > 10. The *Z. bungeana* genes included 3 in the PAL family, 8 in the 4CL or 4CL-like family, and 1 gene each in the C4H, CHS, CHI, FNSII, FLS, DFR (dihydroflavonol reductase), and ANS family. Interestingly, unigenes c334398_g1, c378458_g1, and c332631_g1 were specifically expressed in the inflorescence, and encoded CHS, CHI, and ANS, respectively. Specific primers were designed to amplify the cDNAs of the 18 candidate gene sequences via RT-PCR. Based on the PCR amplification and cDNA sequencing results, the coding sequences of 14 genes were confirmed and the sequences were submitted to Genbank. The accession numbers of these 14 genes are: MN599456, *Zb4CL1*; MN599457, *Zb4CL2*; MN599458, *Zb4CL3*; MN599459, *Zb4CL4*; MN599460, *Zb4CLK1*; MN599461, *Zb4CLK2*; MN599462, *Zb4CLK3*; MN599463, *Zb4CLK4*; MN599471, *ZbCHS1*; MN599465, *ZbCHI1*; MN599464, *ZbFNSII*; MN599467, *ZbFLS*; MN599466, *ZbDFR*; MN599472, *ZbANS*. The FRKM values of these genes are shown in Additional file 6.

### The phylogenetic relationships of the proteins and tissue-specific expression patterns of the flavonoid biosynthesis genes

**Fig. 5:**
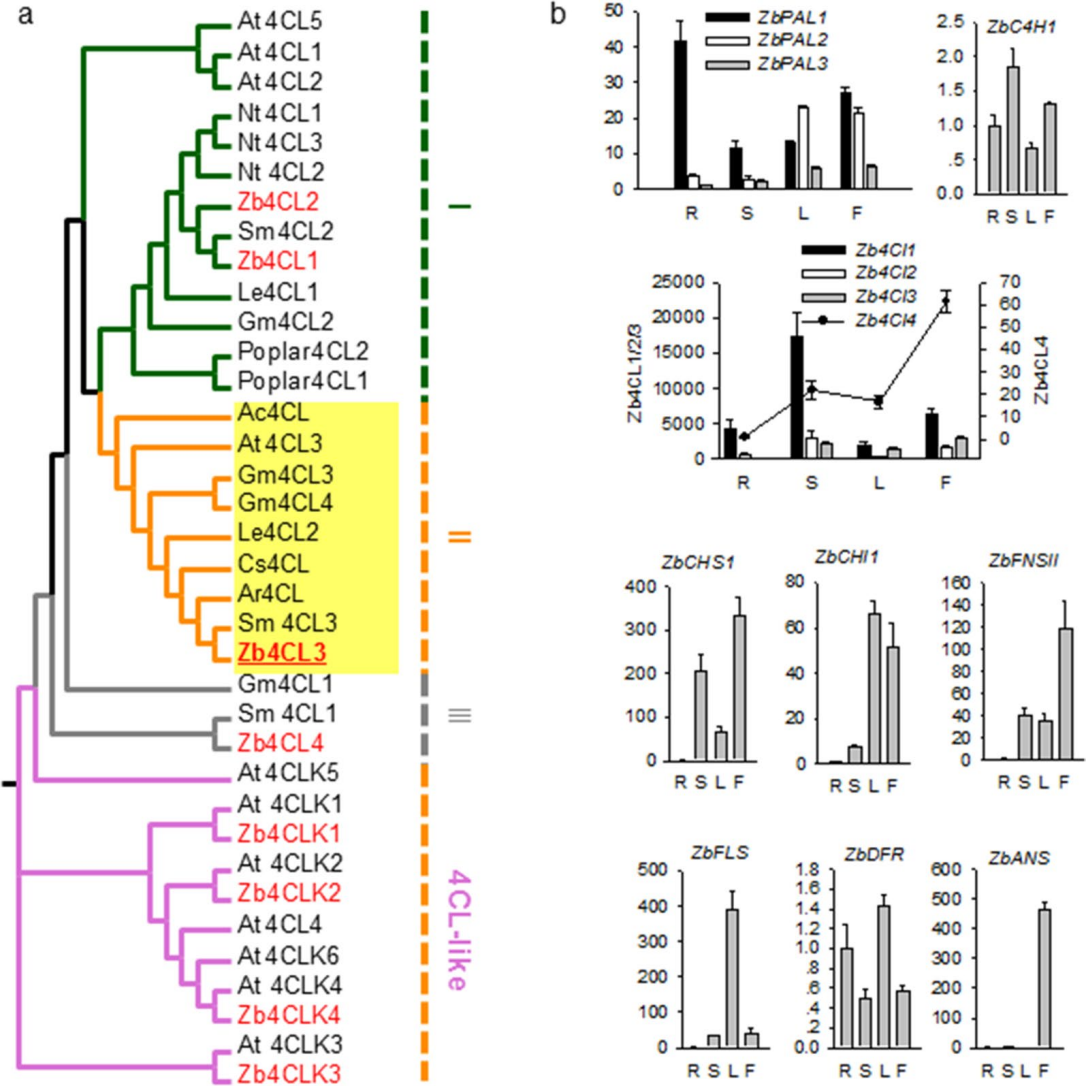
Gene expression pattern and phylogenetic relationship analysis. **a** Phylogenetic relationships for 4CLs and 4CL-likes in *Ziziphora bungeana* and other plant species. Group I 4CLs are highlighted with dark-green branches, putatively participate in lignin synthesis, or the production of additional phenolic compounds other than flavonoids. Group II 4CLs is highlighted with orange branches and yellow leaf background. This group probably participates in flavonoid biosynthesis. Group III is highlighted with grey branches. This group is putatively participated in the biosynthesis of more broad substrates including sinapate. The 4CL-like group is highlighted with orchid branches. The Zb4CLs are heightened with red fonts. The accession numbers of 4CL or 4CL-like sequences used to construct the phylogenetic tree and the names of the species are showed as follows: *Arabidopsis thaliana*, At4CL1 (U18675), At4CL2 (AF106086), At4CL3 (AF106088), At4CL4 (NM_125733.3), At4CL5 (NM_113018.4), At4CLK1 (NM_116755.5), At4CLK2 (NM_118019.3), At4CLK3 (NM_114758.5), At4CLK4 (NM_114758.5), At4CLK4 (NM_101901.4), At4CLK5 (NM_104972.3), At4CLK6 (NM_001344228.1); *Nicotiana tabacum*, Nt4CL1 (D43773.1), Nt4CL2 (U50845.1), Nt4CL3 (U50846.1); *Salvia miltiorrhiza*, Sm4CL1 (AY237163.1), Sm4CL2 (AY237164.1), Sm4CL3 (KF220556); *Agastache rugosa*, Ar4CL (AY587891); *Allium cepa*, Ac4CL (AY541033); *Glycine max*, Gm4CL1 (AF279267), Gm4CL2 (AF002259), Gm4CL3 (AF002258), Gm4CL4 (X69955); *Lithospermum erythrorhizon*, Le4CL1 (D49366), Le4CL2 (D49367); *Camellia sinensis*, Cs4CL (DQ194356); Populus hybrid, Poplar4CL1 (AF008184), Poplar4CL2 (AF008183); *Ziziphora bungeana*, Zb4CL1 (MN599456), Zb4CL2 (MN599457), Zb4CL3 (MN599458), Zb4CL4 (MN599459), Zb4CLK1 (MN599460), Zb4CLK2 (MN599461), Zb4CLK3 (MN599462) and Zb4CLK4 (MN599463). **b** Tissue-specific expression patterns of *ZbPALs*, *Zb4CLs*, *ZbC4H1*, *ZbCHS1*, *ZbCHI1*, and *ZbFNSII* in the root (R), stem (S), leaf (L) and inflorescence (F). The number at the y-axis represents the fold changes of gene expression levels. The relative abundance of *ZbPAL3*, *Zb4CL3*, *ZbC4H1*, *ZbCHS1*, *ZbCHI1*, *ZbFNSII*, *ZbFLS*, *ZbDFR* and *ZbANS* in root is arbitrarily set to 1 in each chart, respectively

4CL (EC 6.2.1.12) is the key enzyme involved in the three steps in the common phenylpropanoid pathway and is the main branch point enzyme generating activated thioesters [56]. The 4CLs from monocot and dicot plants are separated into two distinct clades. In dicot plants, the bona fide 4CLs can be grouped into three clusters; the type I cluster is mainly involved in lignin synthesis or the production of additional phenolic compounds other than flavonoids, the type II cluster is most probably associated with flavonoid biosynthesis [57, 58]; the type III cluster may be involved in converting more broad substrates, including sinapate [59]. The identified 8 genes that encode 4CL and 4CL-like proteins in *Z. bungeana* were used to construct a phylogenetic tree with the 4CL and 4CL-like proteins from *Arabidopsis*, and the bona fide 4CLs from several other plant species (Fig. 5a). The results showed that 4 of the putative Zb4CLs were clustered in 4CL-like groups, which probably have no measurable catalytic activity toward phenylpropanoid pathway intermediates, and so they were named Zb4CLK1, Zb4CLK2, Zb4CLK3, and Zb4CLK4 [60]. For the other bona fide Zb4CLs, Zb4Cl1 and Zb4CL2 were classified as type I 4CLs, Zb4CL3 was classified as a type II 4CL, and Zb4CL4 was classified as a type III 4CL [57, 59, 61]. *Zb4CL1*, *Zb4CL2*, and *Zb4CL4* may also play overlapping roles in flavonoid biosynthesis, like the four isoforms of *4CLs* in *Arabidopsis* [62]. FNSII enzymes play key roles in flavone biosynthesis in plants [63]. The FNSII-1 isoform has more broad specificity for flavanones as substrates, whereas the FNSII-2 isoform is specific for pinocembrin and responsible for the synthesis of chrysin [63–69]. To identify the relationship between FNSIIs in *Z. bungeana* and other plant species, a phylogenetic tree was constructed (Additional file 2: Figure S9). The result showed that ZbFNSII is closely related to CYP93B25 in *Salvia miltiorrhiza*, which suggests that the two proteins may have similar functions.

Based on the predicted sequences of *ZbPAL1*, *ZbPAL2*, *ZbPAL3*, and *ZbC4H1*, and the verified sequences of *Zb4CLs*, *ZbCHS1*, *ZbCHI1*, *ZbFNSII*, *ZbFLS*, *ZbDFR* and *ZbANS*, primers were designed to analyse their expression patterns by qPCR (Fig. 5b). *ZbEF1γ* was used as the internal reference gene; it was confirmed as suitable for qPCR after evaluation with GeNorm, NormFinder, and BestKeeper software (Genbank accession number MN599468) [42]. Among the three *ZbPALs*, qRT-PCR analysis showed that *ZbPAL1* had the highest expression level in roots, *ZbPAL2* had the highest expression level in leaves, and *ZbPAL3* had the highest expression level in leaves and inflorescence. *ZbC4H1* had the highest expression level in the stem. 4CL (EC 6.2.1.12) is the last enzyme in the common phenylpropanoid pathway. Among the 4 bona fide Zb4CLs, *Zb4CL3* had the highest expression level in inflorescence, followed by in stem and leaf, suggesting that it plays essential roles in flavonoid biosynthesis in flowers. *Zb4CL1* and *Zb4CL2* had the highest expression levels in the stem, which suggesting they may be involved in the biosynthesis of lignin and phenolic compounds. The expression level of *Zb4CL4* was the highest in inflorescence. Among the other genes that encode CHS (EC 2.3.1.74), CHI (EC 5.5.1.6), FNSII (EC 1.14.19.76), FLS (EC 1.14.20.6), DFR (EC 1.1.1.219), and ANS (EC 1.14.20.4), *ZbCHS1*, *ZbFNSII*, and *ZbANS* had the highest expression levels in inflorescence, whereas *ZbCHI1*, *ZbFLS*, and ZbDFR had the highest expression levels in leaves (Fig. 5).

## Discussion

### Tissue-specific flavonoid compositions in *Z. bungeana* and the proposed mode of action of linarin for myocardial tissues

This study has revealed 39 chemical compounds from *Z. bungeana* extracts by LC–MS/MS, including 25 flavonoids and 22 possible new compounds. Analysis of the relative redundancies of the 12 major flavonoids (including two new compounds) indicated that 9 flavonoids have tissue-specific distributions. The 9 flavonoids were taken as the characteristic chemical compounds of the root, stem, leaves, or inflorescence. We detected 5 (poly)methoxylated flavones (5, 9, 10, 11, and 12, in Fig. 1). Methoxylated flavones have an array of bioactivities and may be involved in plant chemical defence mechanisms; they also represent promising natural lead molecules for the development of potential antiproliferative, antidiabetic, or anti-inflammatory drugs [70]. In rice, the naringenin *O*-methyltransferase (OsNOMT) can be induced by UV and jasmonate treatment [71]. *Z. bungeana* is distributed in the Tianshan and Altai mountains (altitude 1200–2500 m) in Xinjiang, China, where there is always strong UV radiation in summer. Therefore, we suggest that the (poly)methylation of flavonoids in *Z. bungeana* may be induced by UV radiation or some other adverse factors. Consistent with previous studies, linarin was found to be the major flavonoid component, and it was found to mainly accumulate in flowers [16]. Previous studies also showed that acacetin extracted from *Z. bungeana* has an important protection function for rat cardiomyocytes, but the content of acacetin in the extracts was very low (0.004–0.005%) [13]. In this study, we did not detect any acacetin in *Z. bungeana* extracts. However, it has been reported that linarin is unstable and can be hydrolysed to acacetin in vitro and in the rat intestine [72, 73]. Considering the available evidence, we postulate that linarin can be hydrolysed into acacetin and glycosides in the stomach of rats, and the resulting acacetin has a protective effect on rat myocardial tissue.

### A functional gene analysis platform has been established based on transcriptome profiling and data analysis

In this work, we acquired 397,182 unigenes by transcriptome profiling and data analysis. In these unigenes, 97,562 sequences had lengths between 1000 and 4000 bp. This number is far more than other plant species, such as *Arabidopsis*, rice, or *Salvia miltiorrhiza*. This indicates that the sequencing result has sufficient depth and coverage. Thus, it appears that a combination of Illumina- and SMRT-based RNA sequencing techniques can acquire more transcriptome data with a comparatively lower cost as compared to the use of a single sequencing method. Furthermore, this method is more conducive for identifying full-length cDNAs, alternative-splicing events, and gene expression patterns [74–77]. In this study, besides massive full-length genes, we also discovered alternative-splicing events, such as for some *Zb4CL* genes. Further research is currently underway to identify the underlying causes and modes of action of these alternative transcripts. Until now, the genome of *Z. bungeana* has been unknown; thus, the *Z. bungeana* transcriptome reported here will serve as a valuable resource for further *Z. bungeana* functional gene research.

### Systemic identification of enzyme genes involved in flavonoid biosynthesis pathways

Analysis of the DEGs enriched in GO categories and KEGG pathways, and the BLAST search, identified a total of 18 unigenes with high similarities to enzyme genes involved in flavonoid biosynthesis in other plant species. Among them, the coding sequences of 14 genes were verified by cDNA sequencing, including 8 genes that encode 4CL or 4CL-like proteins. In *Euscaphis konishii* Hayata [77], *Arabidopsis* [78], *Camellia sinensis* [79], and *Ilex paraguariensis* [80], tissue-specific gene expression is closely related with tissue-specific synthesis, accumulation, and modification of flavonoids. Thus, we analysed the relationships between tissue-specific gene expression patterns and flavonoid accumulation in *Z. bungeana*. Phylogenetic analysis revealed that Zb4CL3 may be involved in flavonoid biosynthesis. Tissue-specific expression analysis revealed that *Zb4CL3* has a higher expression level in inflorescence than other tissues, which suggests that it plays a specific role in flavonoid biosynthesis in flowers. However, the other three Zb4CLs may also play overlapping roles in flavonoid biosynthesis, like the four isoforms of 4CLs in *Arabidopsis* [62]. Based on the FPKM value analysis and qPCR analysis, *ZbCHS1* had the highest expression level in inflorescence, suggesting it plays a significant role in the crucial branch point of the flavonoid biosynthesis pathway in the inflorescence. ZbFNSII is closely related to CYP93B25, and *ZbFNSII* had the highest expression level in inflorescence, which suggests that it plays an important role in flavone biosynthesis (Additional file 2: Figure S9 and Fig. 5). *ZbANS* also had a very high expression level in inflorescence, but we did not detect any related anthocyanidin in extracts from various tissues. This may be because anthocyanidins are water-soluble substances and cannot be extracted from 70% ethanol solvent with relatively low polarity [81]. qPCR analysis revealed that *ZbFLS* had the highest expression level in leaves, suggesting that they have specific functions in the biosynthesis of some flavonol glycosides (such as 1 and 3 in Fig. 1) in leaves. In this study, we discovered that most of the flavones in *Z. bungeana* were 7-*O*-rutinosides flavones, which suggests that flavones from different plant species have specific glycosylation modes [81–85]. Based on our comparison of gene expression levels, we suggest that gene expression analysis results based on qPCR are more reliable than those based on FPKM values because qPCR provided better differentiation of gene family members. However, the FPKM value analysis result can provide reference data for the overall range of gene expression levels. A combination of qPCR and FPKM value analysis may produce more reliable results with respect to gene expression levels.

### Proposed biosynthesis pathways of the 12 major flavonoids in *Z. bungeana*

**Fig. 6:**
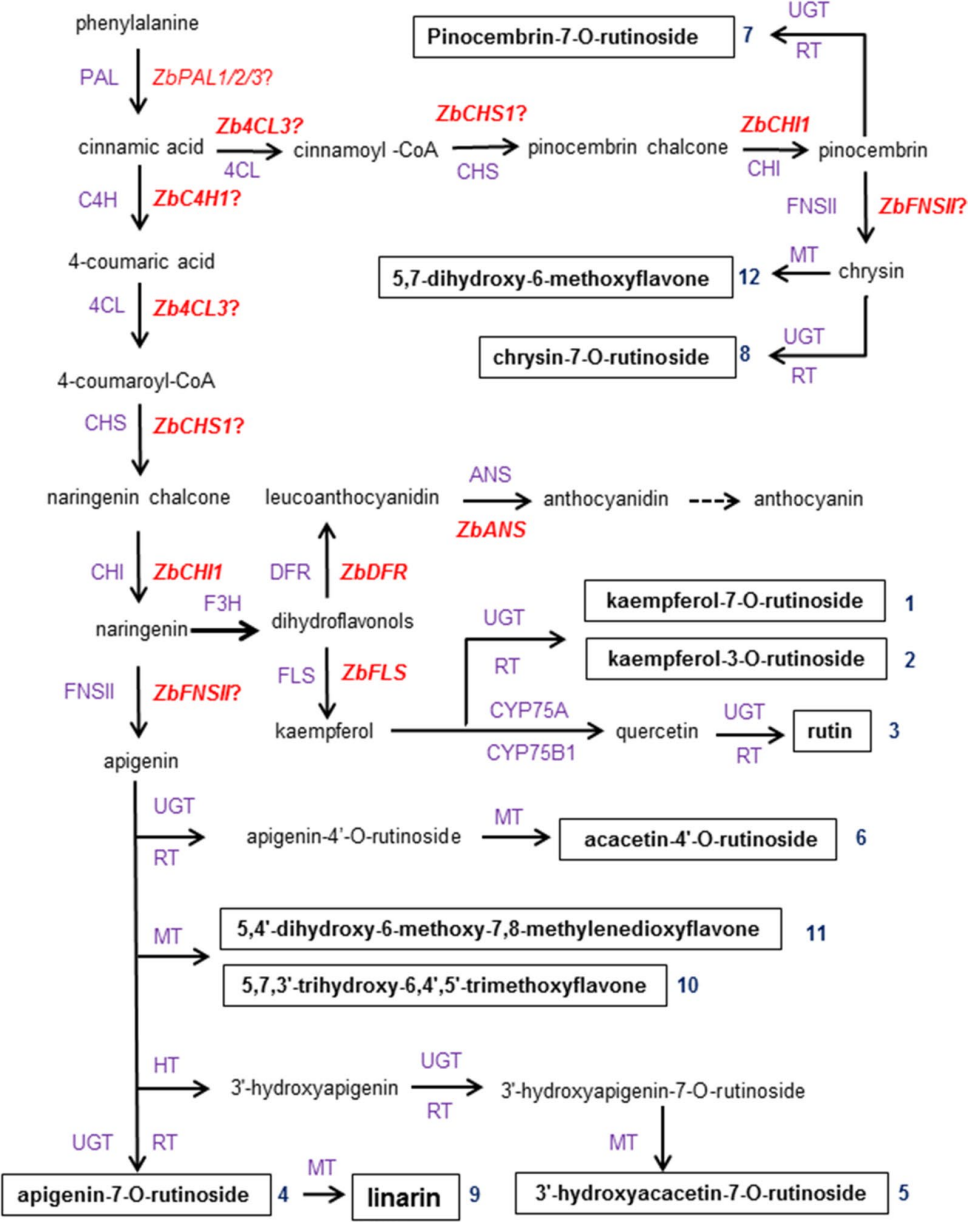
The proposed core pathways responsible for flavonoid biosynthesis in *Z. bungeana*. The full name of the enzymes (violet color font) are: PAL, phenylalanine ammonia-lyase; C4H, cinnamate-4-hydroxylase; 4CL, coumaroyl-CoA ligase; CHS, chalcone synthase; CHI, chalcone isomerase; FNSII, flavone synthase II; F3H, flavanone-3-hydroxylase; DFR, dihydroflavonol reductase; ANS, anthocyanidin synthase; FLS, flavonol synthase; CYP75A, flavonoid 3′,5′-hydroxylase; CYP75B1, flavonoid 3′-monooxygenase; UGT, flavonoid *O*-glycosyltransferase; RT, UDP-rhamnose synthase; HT, hydroxylase; MT, flavonoid *O*-methyltransferase. The candidate genes encoding the enzymes involved in flavonoid biosynthesis are highlighted by red color and italic font, and the identified genes in them are highlighted by bolded font. The identified chemical components are highlighted by bold font and with black text boxes

*Z*. *Bungeana* contains special flavonoids, which may be correlated with its pharmaceutical efficacy. The biosynthesis pathways for some special flavonoids have remained largely unknown. Based on the reported results and our analysis, we have proposed the biosynthesis pathways for 12 major flavonoids including two new compounds in *Z*. *Bungeana* (Fig. 6). The map also depicts the involvement of several new genes. It is proposed that *ZbPAL*s, *Zb4CL3*, *ZbCHS1*, and *ZbCHI1* are involved in the biosynthesis of the flavonoid skeleton; *ZbFNSII* is involved in the accumulation of linarin; and *ZbFLS* is involved in the accumulation of some flavonol glycosides in the leaves of *Z. bungeana*. *ZbANS* is involved in the biosynthesis of anthocyanin. This map provides the first systematic summary of the main flavonoid components, their synthesis pathways, and the involved genes in *Z. bungeana*. It provides a research basis for producing some specific flavonoids of *Z. bungeana* via bio-engineering.

Unlike model plants, the biosynthesis pathways of some secondary metabolites in non-model plants is not single, for example, the proanthocyanins in *Euscaphis konishii* Hayat have two biosynthesis pathways [77]. In the 12 major flavonoids, linarin with the highest relative abundance may be one of the main active ingredients. Recently, the linarin biosynthesis pathway in *C. indicum* has been described. It was suggested linarin is produced by adding a rhamnose to acacetin-7-*O*-glucoside [23]. However, linarin is most probably biosynthesized by adding a methoxyl group to apigenin-7-*O*-rutinoside in *Z*. *Bungeana* because it was identified by LC–MS/MS but not acacetin-7-*O*-glucoside. Moreover, the glycosylated modification of apigenin may occur before the methoxylation step in *Z. bungeana*. These findings have provided new insight into the key steps in linarin biosynthesis.

### Major contributions to resource protection and drug development

Our results showed the tissue-specific distribution of flavonoids in *Z. bungeana*, which supported the use of the aerial parts of the plant for medicine but not for including roots. Since *Z. bungeana* is a kind of perennial plants, retaining its roots will be benefit for its reproduction in the next year. The full-length transcriptome can also contribute to investigate the mechanism of *Z*. *Bungeana*’s growth, development and defense. So this finding is in favor of protecting this medicinal resource. Besides, this study has revealed some new enzyme genes that involved in the biosynthesis pathways of the 12 major flavonoids in this species. These enzyme genes can be introduced into expression platforms to elucidate the functions of the enzymes, which may be used to biosynthesize some drugs or intermediates by bio-engineering. Furthermore, the new flavonoids discovered in *Z*. *Bungeana* will contribute to the natural drug development.

## Conclusions

This study integrated metabolomic and transcriptomic data to analyze the flavonoid constituents and their biosynthesis pathways in *Z. bungeana*. The 12 major flavonoids were revealed by metabolomic data and 9 of these flavonoids were tissue-specific components, of which, linarin was the most abundant flavonoid in *Z. bungeana*. Compared with the whole herb, linarin was abundant in the aerial parts of the plant, especially in flowers, providing a theoretical basis for using the aerial parts of *Z. bungeana* for medicine as possible. Linarin might be primarily biosynthesized from apigenin-7-*O*-rutinoside and hydrolysed into acacetin and glycosides in the stomach of rats, then the resulting acacetin had a protective effect on rat myocardial tissue. Based on the functional gene research platform established by full-length transcriptomic profiling, we have identified 18 unigenes that are probably involved in the core flavonoid biosynthesis pathways in *Z. bungeana* and 7 genes are firstly described as key genes for flavonoid biosynthesis. Finally, the biosynthesis pathway map for the 12 major flavonoids in *Z. Bungeana* was provided. It has provided a basis for the resource protection and drug development from this medicinal plant.

## Supplementary information

**Additional file 1: Table S1.** Primers used to amplify the coding sequences of the unigenes in *Ziziphora bungena*; **Table S2.** qPCR primers used for gene expression analysis; **Table S3.** Identification of flavonoid components in root, stem, leaf, inflorescence of *Ziziphora bungeana* using LC–MS/MS in negative ion and positive ion mode; **Table S4.** Variance analysis of relative peak area of the 12 flavonoids from *Z. bungeana*; **Table S5.** Statistics data for transcriptome; **Table S6.** Summary of the unigene annotation results.

**Additional file 2: Figure S1.** The total ion chromatograms for compounds in different tissues of *Z. bungeana*; **Figure S2.** The heatmap for all the DEGs; **Figure S3.** Volcano plots showed the distribution and fold changes of DEGs in *Z. bungeana*; **Figure S4.** Functional categorization of DEGs of *Z. bungeana* based on GO categories; **Figure S5.** The KEGG enrichment result (Rf *vs*. Ff); **Figure S6.** The KEGG enrichment result (Sf *vs*. Ff); **Figure S7.** The KEGG enrichment result (Lf *vs*. Ff); **Figure S8.** The KEGG enrichment result (Faf *vs*. Ff); **Figure S9.** Phylogenetic relationships for plant FNSII enzymes.

**Additional file 3.** DEGs assigned in GO and KEGG.

**Additional file 4.** Flavonoid biosynthesis related DEGs.

**Additional file 5.** FPKM values of 36 candidate unigenes.

**Additional file 6.** FPKM values of the 14 genes.

## Data Availability

The datasets supporting the conclusions of this article are included within the article and its additional files.

## Abbreviations

HPLC: High-performance liquid chromatography
LC–MS/MS: Liquid chromatography-tandem mass spectrometry
qPCR: Real-time quantitative PCR
PAL: Phenylalanine ammonia-lyase
C4H: Cinnamate-4-hydroxylase
4CL: Coumaroyl-CoA ligase
CHS: Chalcone synthase
CHI: Chalcone isomerase
FNSII: Flavone synthase II
F3H: Flavanone-3-hydroxylase
DFR: Dihydroflavonol reductase
ANS: Anthocyanidin synthase
FLS: Flavonol synthase
CYP75A: Flavonoid 3′,5′-hydroxylase
CYP75B1: Flavonoid 3′-monooxygenase
UGT: Flavonoid *O*-glycosyltransferase
RT: UDP-rhamnose synthase
HT: Hydroxylase
MT: Flavonoid *O*-methyltransferase

## References

[1] Srivedavyasasri R, Zhaparkulova K, Sakipova Z, Ibragimova L, Ross SA (2018). Phytochemical and Biological Studies on *Ziziphora bungeana*. Chem Nat Compd.

[2] Liu Y, Ikem S (1986). Uygur Medicine Record I. Urumqi.

[3] He J, Yang W, Liu C, Tursunj D, Li Y (2016). Herbal textual research on *Ziziphorae herba*. Modern Chin Med.

[4] Shahbazi Y (2017). Chemical compositions, antioxidant and antimicrobial properties of *Ziziphora clinopodioides* Lam. essential oils collected from different parts of Iran. J Food Sci Technol..

[5] Oganesyan G, Galstyan A, Mnatsakanyan V, Paronikyan R, Ter-Zakharyan Y (1991). Phenolic and flavonoid compounds of *Ziziphora clinopodioides*. Chem Nat Compd.

[6] Li G, Meng Q, Wang L, Luo B, Ge Z, Liu W (2015). Chemical constituents from *Ziziphora clinopodioides*. Chin Tradit Herb Drugs..

[7] Shen J, Liu C, Yang W, Chen W (2018). Antioxidant activity of total flavonoids from *Ziziphora clinopodioides*. Nor Horticul..

[8] Zhang X, An D, Zhang H, Ma X (2018). Protective effect of *Ziziphora clinopodioides* flavonoids on human umbilical vein endothelial cells injured by hydrogen Peroxide. Pharmacol Clin Chin Mater Med..

[9] Boniface PK, Elizabeth FI (2019). Flavones as a privileged scaffold in drug discovery: current Developments. Curr Org Synth.

[10] Singh M, Kaur M, Silakari O (2014). Flavones: an important scaffold for medicinal chemistry. Eur J Med Chem.

[11] Senejoux F, Demougeot C, Kerram P, Aisa HA, Berthelot A, Bevalot F (2012). Bioassay-guided isolation of vasorelaxant compounds from *Ziziphora clinopodioides* Lam. (Lamiaceae). Fitoterapia.

[12] Li Q, Tursunj D, Shi C, Heyrulla M, Zhang X, Yang W (2018). *Ziziphora clinopodioides* flavonoids protect myocardial cell damage from myocardial ischemia-reperfusion injury. Evid Based Complement Altern Med..

[13] Yang W, Liu C, Gu Z, Zhang X, Cheng B, Mao Y (2014). Protective effects of acacetin isolated from *Ziziphora clinopodioides* Lam. (Xintahua) on neonatal rat cardiomyocytes. Chin Med..

[14] Yu Q, Li X, Cao X (2017). Linarin could protect myocardial tissue from the injury of ischemia-reperfusion through activating Nrf-2. Biomed Pharmacother.

[15] Tursun D, He J, Harrulla M, Cheng B, Yang W, Aliaji D (2018). Determination of 3 components in Xinjiang *Ziziphora bungeana* by HPLC. China J Chin Mater Med..

[16] He J, Ma Y, Yang W, Cheng B, Tursunj D, Ma L (2019). Transcriptome analysis reveals candidate genes involved in flavonoid biosynthesis in *Ziziphora bungeana*. China J Chin Mater Med..

[17] Tohge T, de Souza LP, Fernie AR (2017). Current understanding of the pathways of flavonoid biosynthesis in model and crop plants. J Exp Bot.

[18] Zhang L, Su W, Tao R, Zhang W, Chen J, Wu P (2017). RNA sequencing provides insights into the evolution of lettuce and the regulation of flavonoid biosynthesis. Nat Commun..

[19] Liu L, Li Y, She G, Zhang X, Jordan B, Chen Q (2018). Metabolite profiling and transcriptomic analyses reveal an essential role of UVR8-mediated signal transduction pathway in regulating flavonoid biosynthesis in tea plants (*Camellia sinensis*) in response to shading. BMC Plant Biol.

[20] Deng Y, Li C, Li H, Lu S (2018). Identification and characterization of flavonoid biosynthetic enzyme genes in *Salvia miltiorrhiza* (Lamiaceae). Molecules.

[21] Deshmukh AB, Datir S, Bhonde Y, Kelkar N, Samdani P (2018). De novo root transcriptome of a medicinally important rare tree *Oroxylum indicum* for characterization of the flavonoid biosynthesis pathway. Phytochemistry.

[22] Yue J, Zhu C, Zhou Y, Niu X, Miao M, Tang X (2018). Transcriptome analysis of differentially expressed unigenes involved in flavonoid biosynthesis during flower development of *Chrysanthemum morifolium* ‘Chuju’. Sci Rep..

[23] Jiang Y, Ji X, Duan L, Ye P, Yang J, Zhan R (2019). Gene mining and identification of a flavone synthase II involved in flavones biosynthesis by transcriptomic analysis and targeted flavonoid profiling in *Chrysanthemum indicum* L. Ind Crops Products..

[24] Zhao Q, Zhang Y, Wang G, Hill L, Weng J, Chen X (2016). A specialized flavone biosynthetic pathway has evolved in the medicinal plant, *Scutellaria baicalensis*. Sci Adv.

[25] Li Q, Li Y, Song J, Xu H, Xu J, Zhu Y (2014). High-accuracy de novo assembly and SNP detection of chloroplast genomes using a SMRT circular consensus sequencing strategy. New Phytol.

[26] Chen J, Tang X, Ren C, Wei B, Wu Y, Wu Q (2018). Full-length transcriptome sequences and the identification of putative genes for flavonoid biosynthesis in safflower. BMC Genomics..

[27] Grabherr M, Haas B, Yassour M, Levin J, Thompson D, Amit I (2011). Full-length transcriptome assembly from RNA-Seq data without a reference genome. Nat Biotechnol.

[28] Xu Z, Peters R, Weirather J, Luo H, Liao B, Zhang X (2015). Full-length transcriptome sequences and splice variants obtained by a combination of sequencing platforms applied to different root tissues of *Salvia miltiorrhiza* and tanshinone biosynthesis. Plant J..

[29] Liao JJ. Study on extraction and purification of total flavonoids from *Ziziphora clinopodioides* Lam. and the preparation technology of dropping pill [dissertation]. Xinjiang Medicinal University; 2012.

[30] Yan G, Sun H, Sun W, Zhao L, Meng X, Wang X (2010). Rapid and global detection and characterization of *aconitum* alkaloids in Yin Chen Si Ni Tang a traditional Chinese medical formula by ultra performance liquid chromatography-high resolution mass spectrometry and automated data analysis. J Pharm Biomed Anal.

[31] Cao G, Zhang Y, Feng J, Cai H, Zhang C, Ding M (2011). A rapid and sensitive assay for determining the main components in processed *Fructus corni* by UPLC-Q-TOF-MS. Chromatographia.

[32] Deng P, You T, Chen X, Yuan T, Huang H, Zhong D (2011). Identification of amiodarone metabolites in human bile by ultraperformance liquid chromatography/quadrupole time-of-flight mass spectrometry. Drug Metab Dispos.

[33] Montoro P, Teyeb H, Masullo M, Mari A, Douki W, Piacente S (2013). LC-ESI-MS quali-quantitative determination of phenolic constituents in different parts of wild and cultivated *Astragalus gombiformis*. J Pharm Biomed Anal.

[34] Zhang Y, Zhang A, Zhang Y, Sun H, Meng X, Yan G (2016). Application of ultra-performance liquid chromatography with time-of-flight mass spectrometry for the rapid analysis of constituents and metabolites from the extracts of *Acanthopanax senticosus* Harms leaf. Pharmacogn Mag..

[35] Fu L, Niu B, Zhu Z, Wu S, Li W (2012). CD-HIT: accelerated for clustering the next-generation sequencing data. Bioinformatics.

[36] Li B, Dewey CN (2011). RSEM: accurate transcript quantification from RNA-Seq data with or without a reference genome. BMC Bioinform..

[37] Ghosh S, Chan CK (2016). Analysis of RNA-Seq data using TopHat and Cufflinks. Methods Mol Biol.

[38] Anders S, Huber W (2010). Differential expression analysis for sequence count data. Genome Biol.

[39] Lu G, Moriyama EN (2004). Vector NTI a balanced all-in-one sequence analysis suite. Brief Bioinform.

[40] Kumar S, Stecher G, Tamura K (2016). MEGA7: molecular evolutionary genetics analysis version 7.0 for bigger datasets. Mol Biol Evol..

[41] He Z, Zhang H, Gao S, Lercher M, Chen W, Hu S (2016). Evolview v2: an online visualization and management tool for customized and annotated phylogenetic trees. Nucleic Acids Res.

[42] Wang Z, Ma Y, Li Y, He J, Yang W (2020). Selection and validation of appropriate reference genes for real-time quantitative RT-PCR analysis in *Ziziphora bungeana* Juz. J Chin Med Mater..

[43] Murata T, Sasaki K, Sato K, Yoshizaki F, Yamada H, Mutoh H (2009). Matrix metalloproteinase-2 inhibitors from *Clinopodium chinense* var. parviflorum. J Nat Prod..

[44] Dawidowicz AL, Bernacik K, Typek R (2018). Umbelliferone instability during an analysis involving its extraction process. Monatsh Chem.

[45] Li G, Meng Q, Luo B, Ge Z, Liu W (2015). Isolation of chemical constituents from *Ziziphora clinopodioides* Lam. with recycling preparative high performance liquid chromatography. Se Pu..

[46] Adizov SM, Mukhamathanova RF, Turgunov KK, Shamyanov ID, Tashkhodjaev B (2013). 5,7-Dihy-droxy-2-(3-hy-droxy-4,5-dimeth-oxy-phen-yl)-6-meth-oxy-4H-chromen-4-one. Acta Crystallogr Sect E Struct Rep Online..

[47] Yang W, Gu Z, Hairulla M, Zhao J, Shi F, He J (2011). Study on flavonoids constituents from *Ziziphora clinopodioides* Lam. Lishizhen Med Mater Med Res.

[48] Zou G, Su Z, Zhang H, Wang Y, Yang JS, Zou Z (2010). Flavonoids from the stems of *Croton caudatus* Geisel. var. *tomentosus* Hook. Molecules..

[49] Shie J, Chen C, Lin C, Ku A, Cheng T, Fang J (2010). Regioselective synthesis of di-C-glycosylflavones possessing anti-inflammation activities. Org Biomol Chem.

[50] Sadhu S, Hirata K, Li X, Ohtsuki T, Koyano T, Preeprame S (2006). Flavonoids and sesquiterpenoids constituents from *Eupatorium capillifolium* found in a screening study guided by cell growth inhibitory activity. J Nat Med.

[51] Yim S, Kim H, Lee I (2003). A polyacetylene and flavonoids from *Cirsium rhinoceros*. Arch Pharm Res..

[52] Edenharder R, Keller G, Platt KL, Unger KK (2001). Isolation and characterization of structurally novel antimutagenic flavonoids from spinach (*Spinacia oleracea*). J Agric Food Chem.

[53] Aquino R, Ciavatta M, De SF (1990). A flavanone glycoside from *Hamelia patens*. Phytochemistry.

[54] Aurnhammer G, Wagner H, Horhammer L, Farkas L (1970). Idenity of the flavanone rhamnoglucosides sarotsnoside and isosarotanoside. Synthesis of pinocombrin-7-beta-neohesperidoside. Chem Ber..

[55] Zhang X, An D, Guo L, Yang N, Zhang H (2019). Identification and screening of active components from *Ziziphora clinopodioides* Lam. in regulating autophagy. Nat Prod Res..

[56] Vogt T (2010). Phenylpropanoid biosynthesis. Mol Plant..

[57] Hamberger B, Hahlbrock K (2004). The 4-coumarate: CoA ligase gene family in *Arabidopsis thaliana* comprises one rare sinapate-activating and three commonly occurring isoenzymes. Proc Natl Acad Sci USA.

[58] Ehlting J, Buttner D, Wang Q, Douglas CJ, Somssich IE, Kombrink E (1999). Three 4-coumarate:coenzyme A ligases in *Arabidopsis thaliana* represent two evolutionarily divergent classes in angiosperms. Plant J..

[59] Wang B, Sun W, Li Q, Li Y, Luo H, Song J (2015). Genome-wide identification of phenolic acid biosynthetic genes in *Salvia miltiorrhiza*. Planta.

[60] Costa MA, Bedgar DL, Moinuddin SG, Kim KW, Cardenas CL, Cochrane FC (2005). Characterization in vitro and in vivo of the putative multigene 4-coumarate:CoA ligase network in *Arabidopsis*: syringyl lignin and sinapate/sinapyl alcohol derivative formation. Phytochemistry.

[61] Lindermayr C, Mollers B, Fliegmann J, Uhlmann A, Lottspeich F, Meimberg H (2002). Divergent members of a soybean (*Glycine max* L) 4-coumarate:coenzyme A ligase gene family. Eur J Biochem..

[62] Li Y, Kim JI, Pysh L, Chapple C (2015). Four isoforms of *Arabidopsis* 4-Coumarate:CoA ligase have overlapping yet distinct roles in phenylpropanoid metabolism. Plant Physiol.

[63] Akashi T, Fukuchi-Mizutani M, Aoki T, Ueyama Y, Yonekura-Sakakibara K, Tanaka Y (1999). Molecular cloning and biochemical characterization of a novel cytochrome P450 flavone synthase II that catalyzes direct conversion of flavanones to flavones. Plant Cell Physiol.

[64] Kitada C, Gong Z, Tanaka Y, Yamazaki M, Saito K (2001). Differential expression of two cytochrome P450s involved in the biosynthesis of flavones and anthocyanins in chemo-varietal forms of *Perilla frutescens*. Plant Cell Physiol.

[65] Martens S, Forkmann G (1999). Cloning and expression of flavone synthase II from *Gerbera hybrids*. Plant J..

[66] Zhao Q, Zhang Y, Wang G, Hill L, Weng JK, Chen XY (2016). A specialized flavone biosynthetic pathway has evolved in the medicinal plant *Scutellaria baicalensis*. Sci Adv..

[67] Wu J, Wang X, Liu Y, Du H, Shu Q, Su S (2016). Flavone synthases from *Lonicera japonica* and *L. macranthoides* reveal differential flavone accumulation. Sci Rep..

[68] Fliegmann J, Furtwangler K, Malterer G, Cantarello C, Schuler G, Ebel J (2010). Flavone synthase II (CYP93B16) from soybean (*Glycine max* L.). Phytochemistry..

[69] Yan J, Wang B, Jiang Y, Cheng L, Wu T (2014). GmFNSII-controlled soybean flavone metabolism responds to abiotic stresses and regulates plant salt tolerance. Plant Cell Physiol.

[70] Anna B, David RG (2016). Methoxylated flavones: occurrence importance biosynthesis. Phytochem Rev.

[71] Shimizu T, Lin F, Hasegawa M, Okada K, Nojiri H, Yamane H (2012). Purification and identification of naringenin 7-O-methyltransferase a key enzyme in biosynthesis of flavonoid phytoalexin sakuranetin in rice. J Biol Chem.

[72] Deng H, Pan H, Chen Z, Zhang Y, Wang L (2016). Modeling of thermal degradation of linarin during concentration process. China J Chin Mater Med..

[73] Feng X, Li Y, Guang C, Qiao M, Wang T, Chai L (2018). Characterization of the in vivo and in vitro metabolites of linarin in rat biosamples and intestinal flora using ultra-high performance liquid chromatography coupled with quadrupole time-of-flight tandem mass spectrometry. Molecules.

[74] Zhu C, Li X, Zheng J (2018). Transcriptome profiling using Illumina- and SMRT-based RNA-seq of hot pepper for in-depth understanding of genes involved in CMV infection. Gene.

[75] Ding N, Cui H, Miao Y, Tang J, Cao Q, Luo Y (2019). Single-molecule real-time sequencing identifies massive full-length cDNAs and alternative-splicing events that facilitate comparative and functional genomics study in the hexaploid crop sweet potato. PeerJ..

[76] Wang L, Jiang X, Wang L, Wang W, Fu C, Yan X (2019). A survey of transcriptome complexity using PacBio single-molecule real-time analysis combined with Illumina RNA sequencing for a better understanding of ricinoleic acid biosynthesis in *Ricinus communis*. BMC Genomics..

[77] Liang W, Ni L, Carballar-Lejarazu R, Zou X, Sun W, Wu L (2019). Comparative transcriptome among *Euscaphis konishii* Hayata tissues and analysis of genes involved in flavonoid biosynthesis and accumulation. BMC Genomics..

[78] Kleindt CK, Stracke R, Mehrtens F, Weisshaar B (2010). Expression analysis of flavonoid biosynthesis genes during *Arabidopsis thaliana* silique and seed development with a primary focus on the proanthocyanidin biosynthetic pathway. BMC Res Notes..

[79] Li CF, Zhu Y, Yu Y, Zhao QY, Wang SJ, Wang XC (2015). Global transcriptome and gene regulation network for secondary metabolite biosynthesis of tea plant (*Camellia sinensis*). BMC Genomics..

[80] Fay JV, Watkins CJ, Shrestha RK, Litwiniuk SL, Talavera Stefani LN, Rojas CA (2018). Yerba mate (*Ilex paraguariensis* A. St.-Hil.) de novo transcriptome assembly based on tissue specific genomic expression profiles. BMC Genomics..

[81] Silva S, Costa EM, Calhau C, Morais RM, Pintado ME (2017). Anthocyanin extraction from plant tissues: a review. Crit Rev Food Sci Nutr.

[82] Crozier A, Jaganath I, Clifford MN, Clifford MN, Ashihara H (2006). Phenols Polyphenols and Tannins: An Overview. Plant secondary metabolites: occurrence structure and role in the human diet crozier A.

[83] Herrmann K (1976). Flavonols and flavones in food plants: a review. J Food Technol.

[84] Crozier A, Yokota T, Jaganath IB, Marks SC, Saltmarsh M, Clifford MN (2007). Secondary metabolites in fruits vegetables beverages and other plant-based dietary components.

[85] Wang M, Simon J, Aviles IF, He K, Zheng QY, Tadmor Y (2003). Analysis of antioxidative phenolic compounds in artichoke (*Cynara scolymus* L). J Agric Food Chem..

