# Supplementary material for: Integrated metabolomic and transcriptomic profiling reveals the tissue-specific flavonoid compositions and their biosynthesis pathways in Ziziphora bungeana
Source: Chin Med. 2020 Jul 16;15:73. doi: 10.1186/s13020-020-00354-6 (PMC7364582; doi:10.1186/s13020-020-00354-6)
Supplement: Supplementary file 2 — Additional file 2: Figure S1. The total ion chromatograms for compounds in different tissues of Z. bungeana; Figure S2. The heatmap for all the DEGs; Figure S3. Volcano plots showed the distribution and fold changes of DEGs in Z. bungeana; Figure S4. Functional categorization of DEGs of Z. bungeana based on GO categories; Figure S5. The KEGG enrichment result (Rf vs. Ff); Figure S6. The KEGG enrichment result (Sf vs. Ff); Figure S7. The KEGG enrichment result (Lf vs. Ff); Figure S8. The KEGG enrichment result (Faf vs. Ff); Figure S9. Phylogenetic relationships for plant FNSII enzymes. [file 13020_2020_354_MOESM2_ESM.docx]

Additional Figures


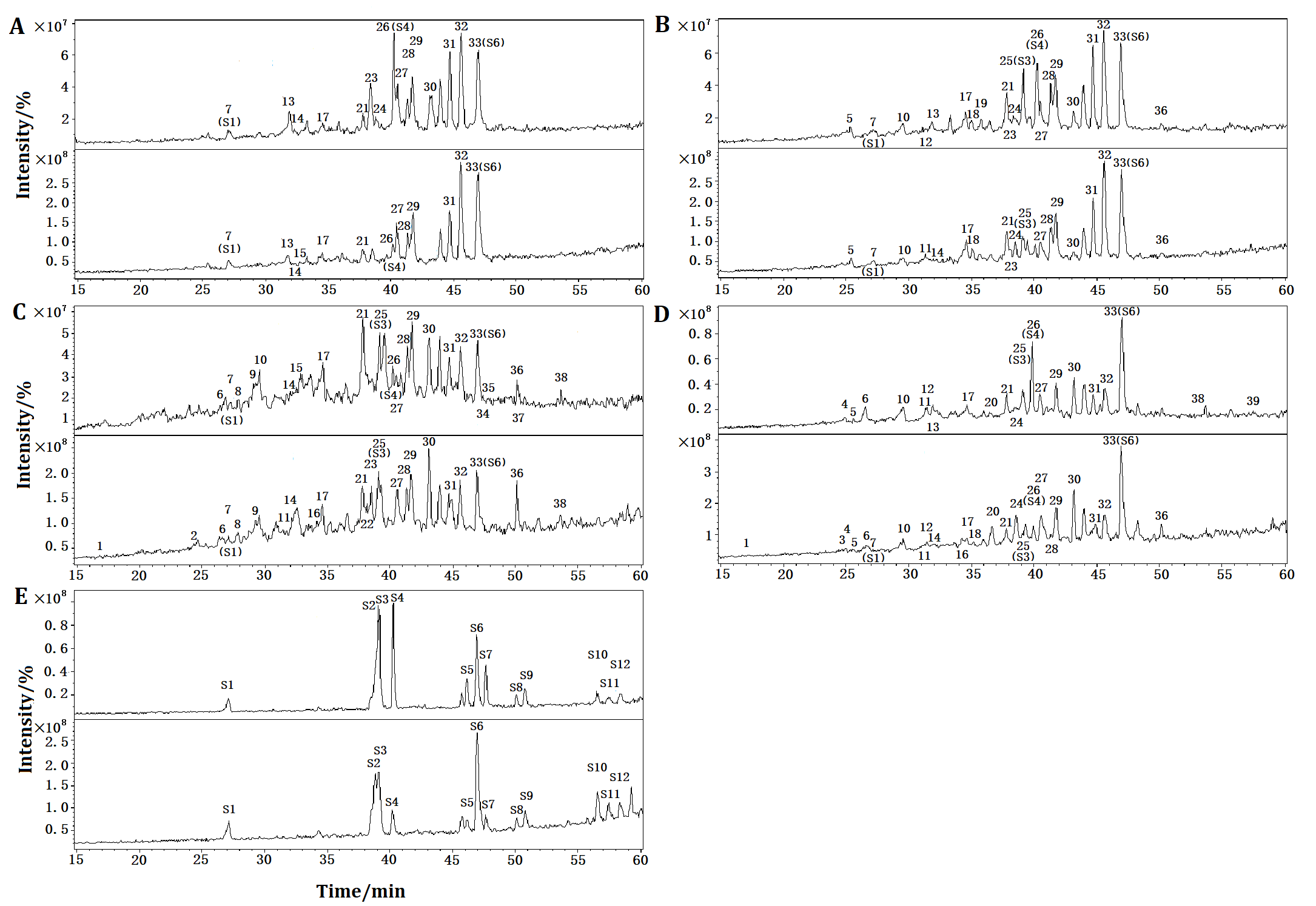


Figure S1. The total ion chromatograms for compounds in different tissues of *Z. bungeana*. A, B, C, D, and E represent root, stem, leaf, inflorescence, and standard compounds, respectively. 1-39，S1-S12 are listed in Table S3 in additional file 1.


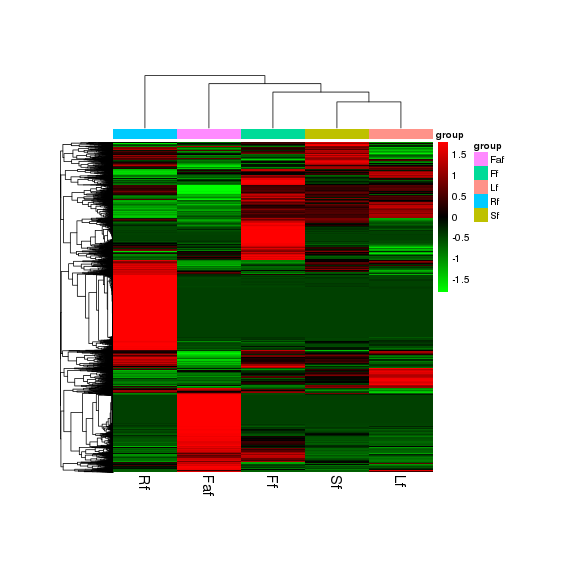


Figure S2. The heatmap for all the DEGs. In the heatmap, the colors from green to red represent the FRKM value, as indicated by the numbers in the ruler. Rf, Sf, Lf, Ff, and Faf represent root, stem, leaf, inflorescence, and infructescence, respectively.


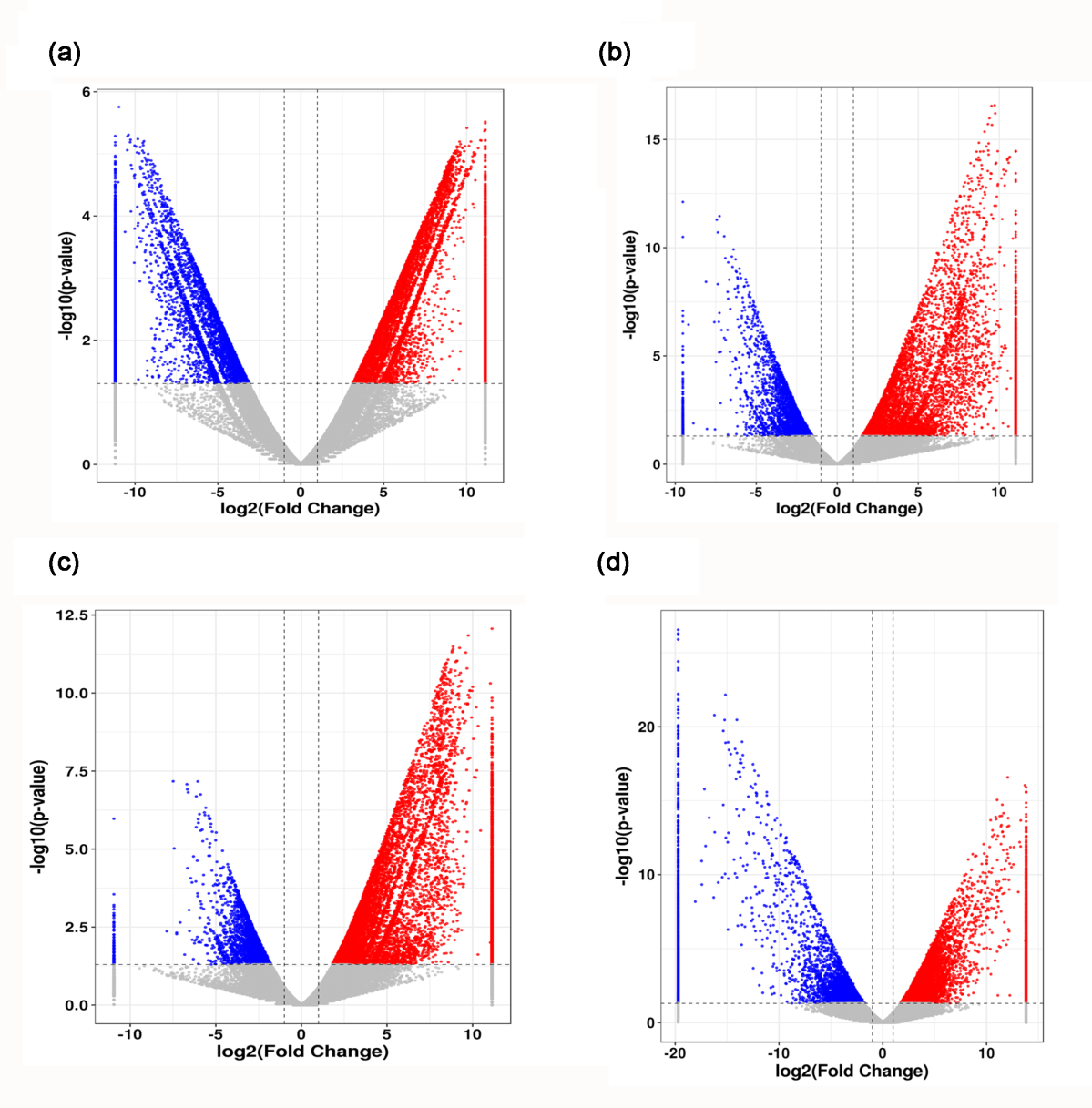


**Figure S3.** Volcano plots showed the distribution and fold changes of DEGs in *Z. bungeana*. (**a**) Root *vs*. inflorescence. The number of down-regulated DEGs is 11,686; the up-regulated is 8,708, and 231,031 DEGs are with no significant difference. (**b**) Stem *vs*. inflorescence. The number of down-regulated DEGs is 3,659, the number of up-regulated is 8,097, and 162,411 DEGs is with no significant difference. (**c**) Leaf *vs*. inflorescence. The number of down-regulated DEGs is 4,083, the number of up-regulated is 10,673, and 150,799 is with no significant difference. (**d**) Infructescence *vs*. inflorescence. The number of down-regulated DEGs is 8,504, the number of up-regulated is 10,267, and 184,607 is with no significant difference.


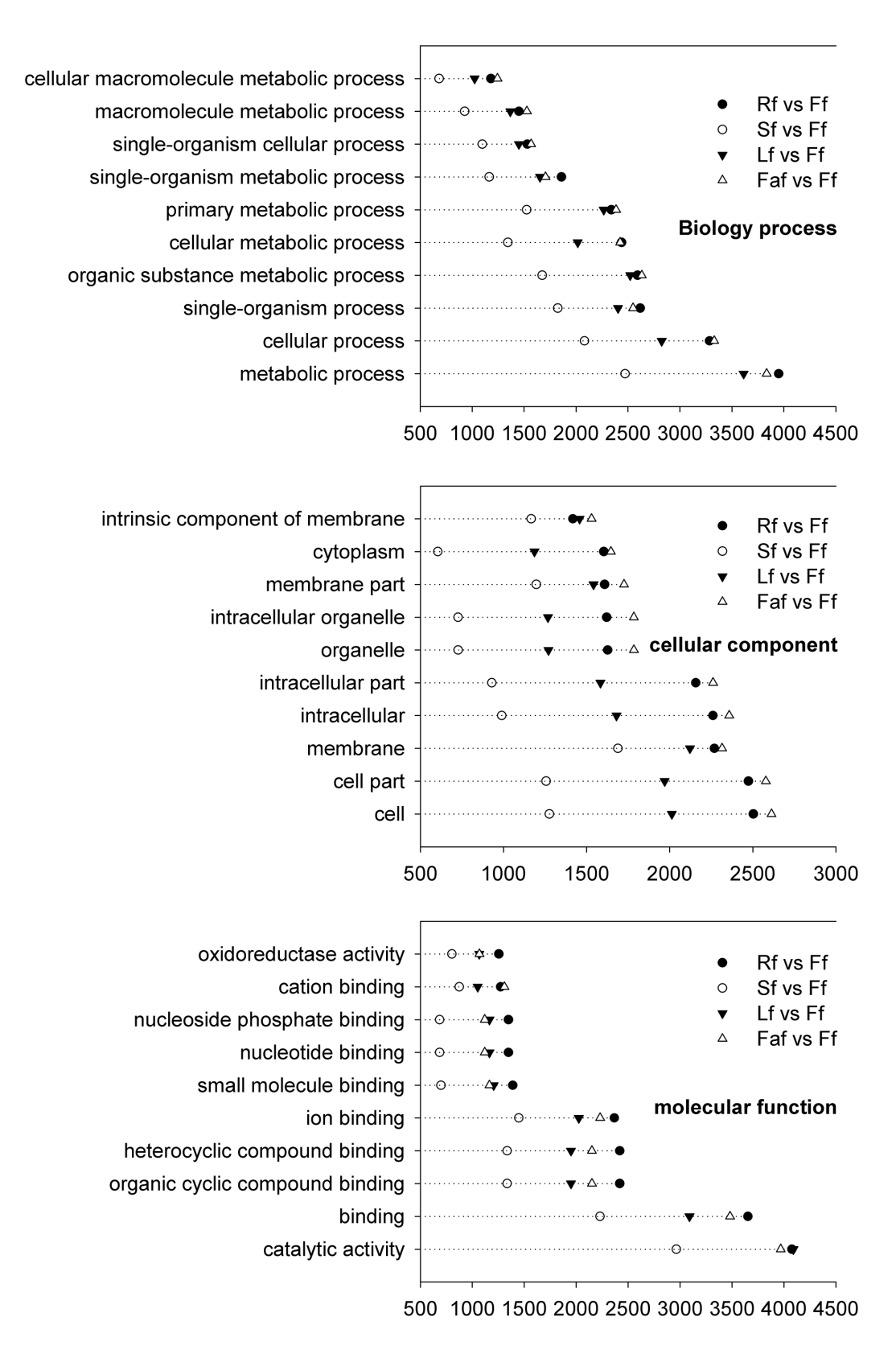


**Figure S4.** Functional categorization of DEGs of *Z. bungeana* based on GO categories. The vertical coordinate represents the most enriched ten GO terms, and the horizontal coordinate represents the number of enriched DEGs.


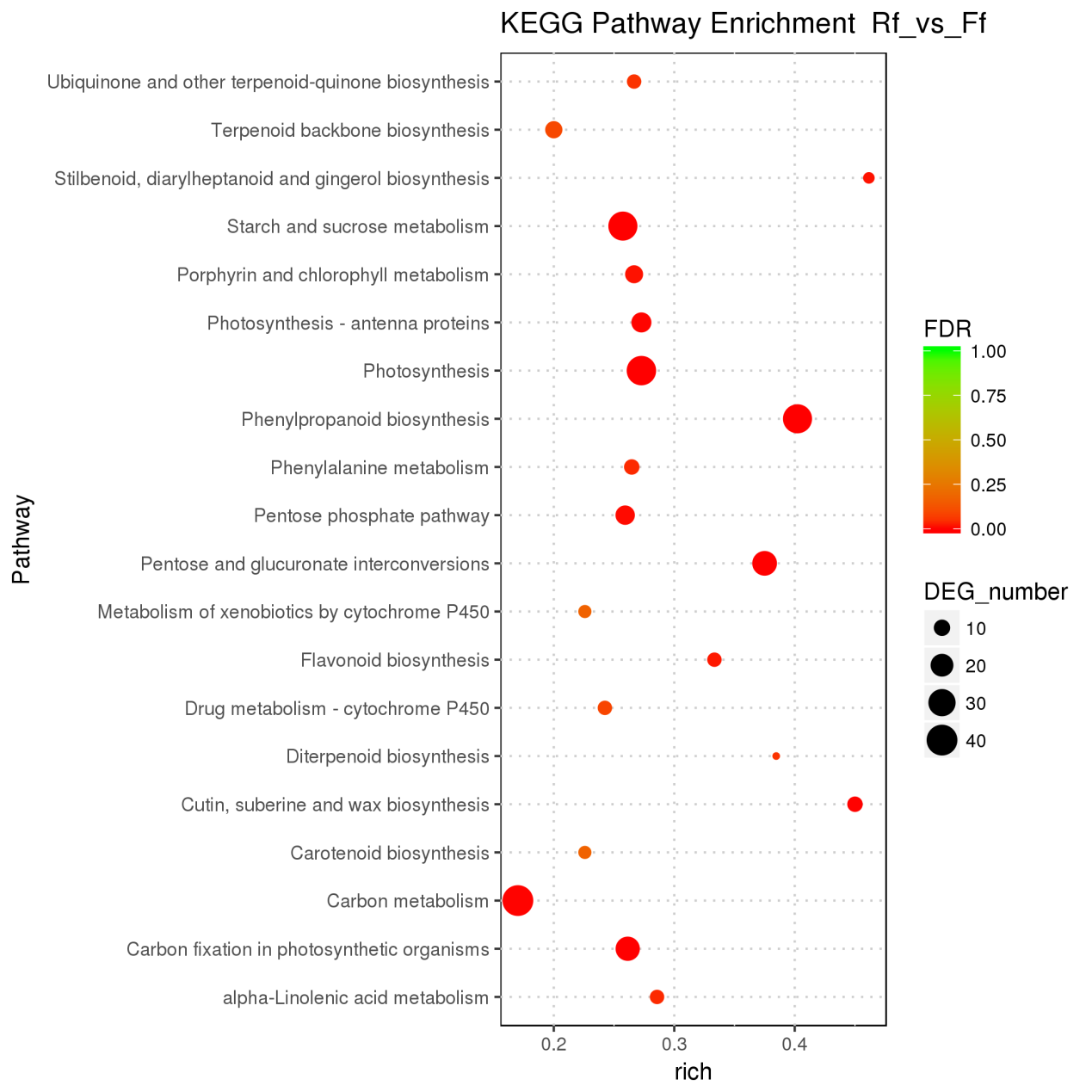


Figure S5. The KEGG enrichment result (Rf vs. Ff). The vertical coordinate is KEGG Pathway entry, and the horizontal coordinate is the rich factor. The size of the dots represents the ratio of the DEGs enriched to all the genes annotated in the pathway. The color represents the P value of the pathway and represents the enrichment significance. The smaller the P value, the more significant is the enriched. The figure shows the 20 most significant enriched paths.


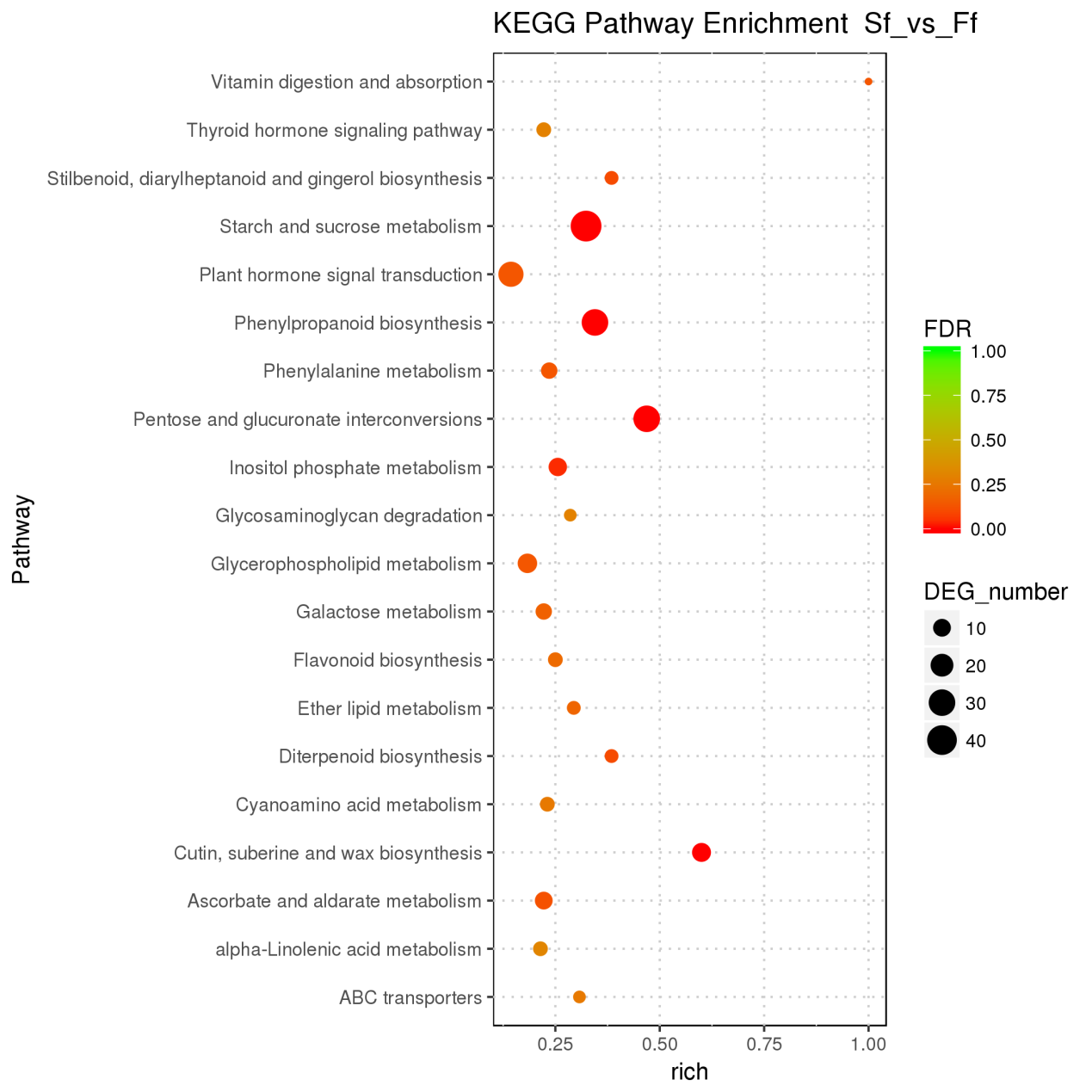


Figure S6. The KEGG enrichment result (Sf vs. Ff). The vertical coordinate is KEGG Pathway entry, and the horizontal coordinate is the rich factor. The size of the dots represents the ratio of the DEGs enriched to all the genes annotated in the pathway. The color represents the P value of the pathway and represents the enrichment significance. The smaller the P value, the more significant is the enriched. The figure shows the 20 most significant enriched paths.


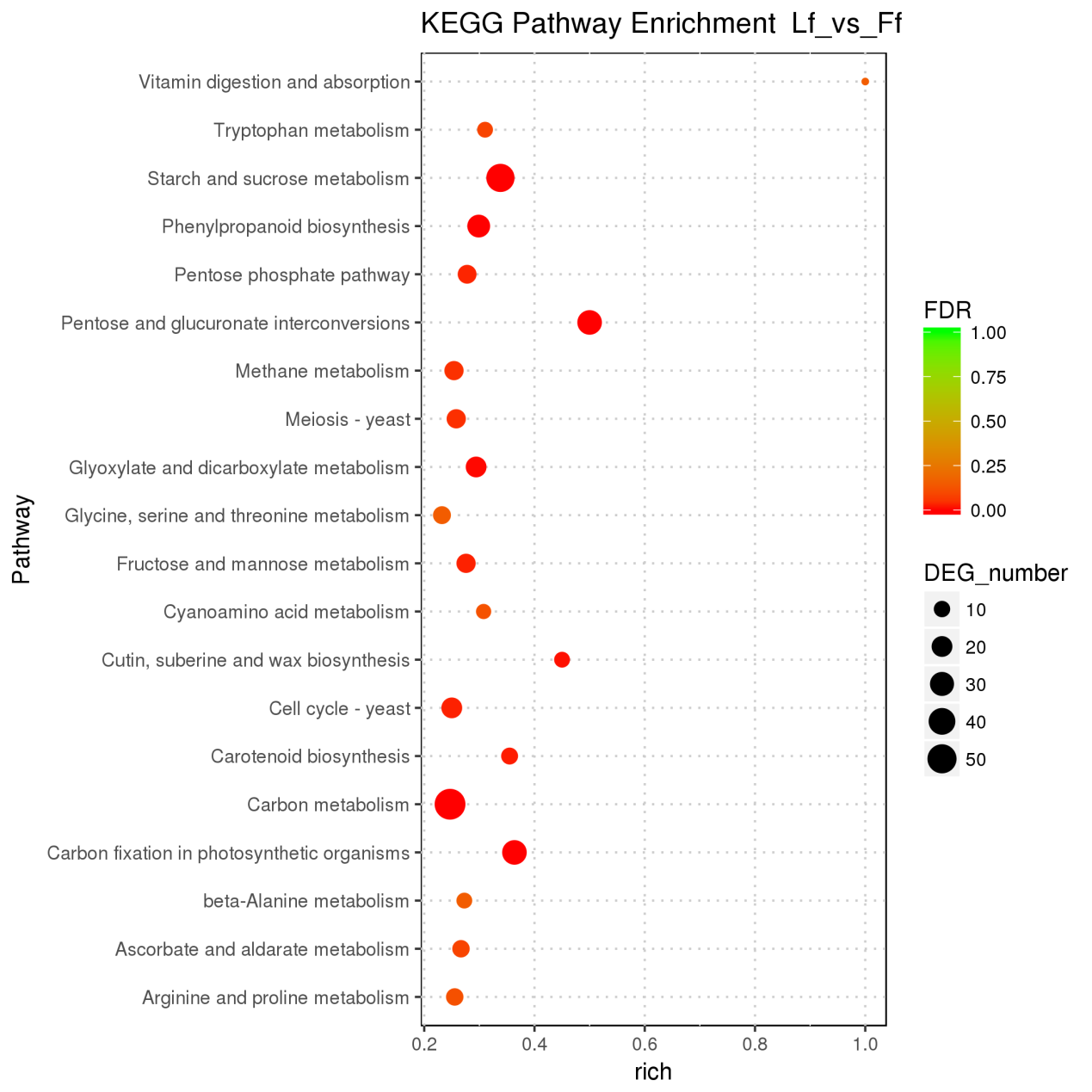


**Figure S7.** The KEGG enrichment result (Lf vs. Ff). The vertical coordinate is KEGG Pathway entry, and the horizontal coordinate is the rich factor. The size of the dots represents the ratio of the DEGs enriched to all the genes annotated in the pathway. The color represents the P value of the pathway and represents the enrichment significance. The smaller the P value, the more significant is the enriched. The figure shows the 20 most significant enriched paths.


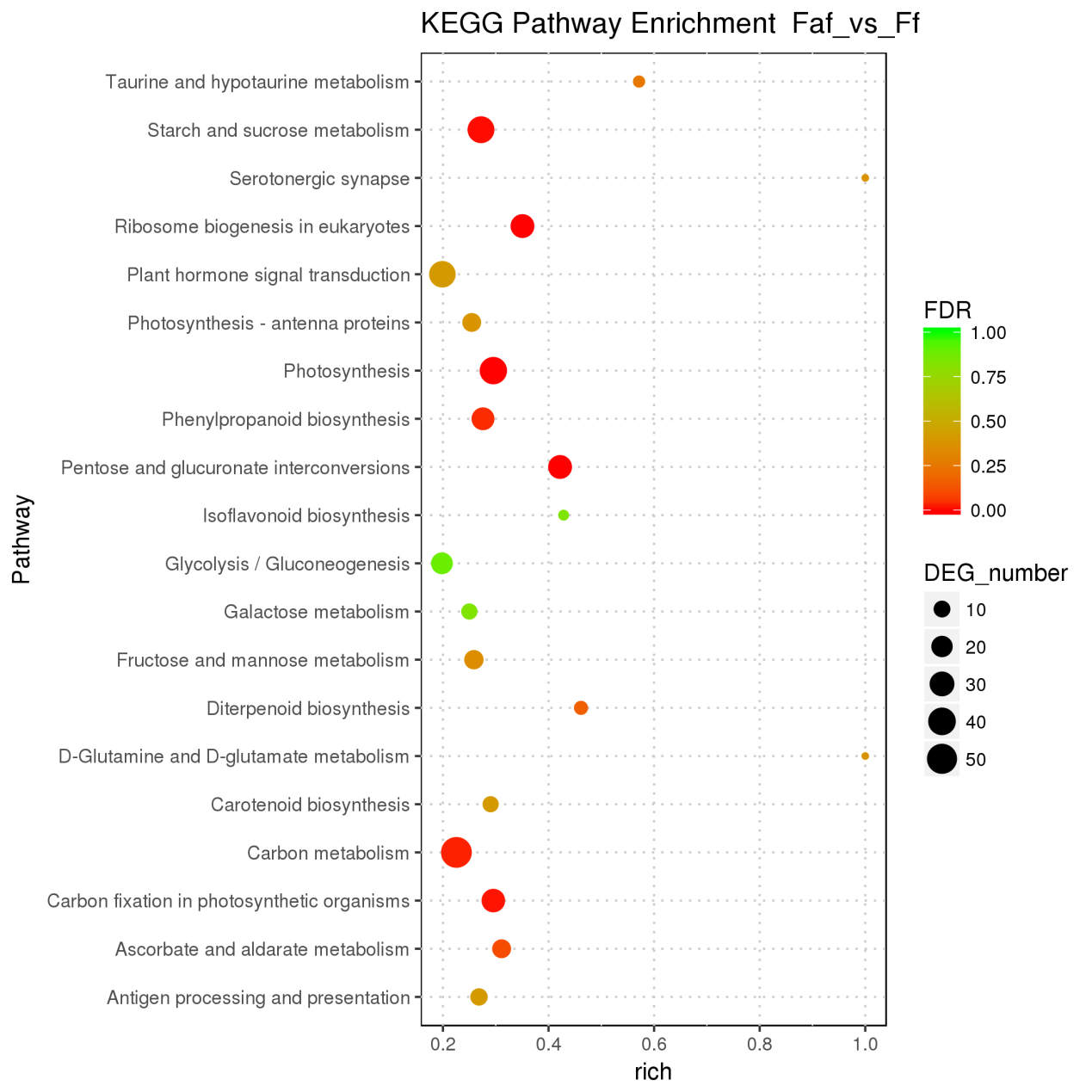


Figure S8. The KEGG enrichment result (Faf vs. Ff). The vertical coordinate is KEGG Pathway entry, and the horizontal coordinate is the rich factor. The size of the dots represents the ratio of the DEGs enriched to all the genes annotated in the pathway. The color represents the P value of the pathway and represents the enrichment significance. The smaller the P value, the more significant is the enriched. The figure shows the 20 most significant enriched paths.


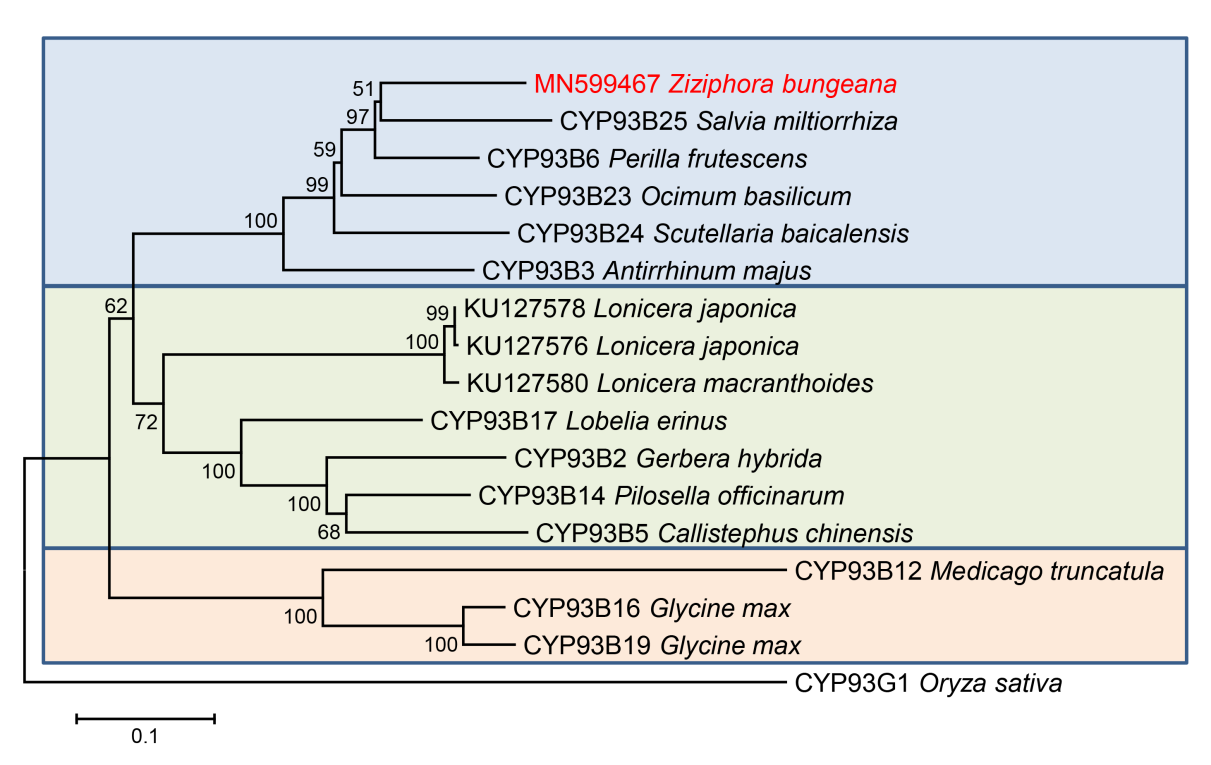


Figure S9. Phylogenetic relationships for plant FNSII enzymes. The tree was rooted with *Oryza sativa* CYP93G1. GenBank ID of the CYP93Bs used in the tree: CYP93G1, AK100972; CYP93B19, NP_001241129.1; CYP93B16, ACV65037.1; CYP93B12, ABC59104.2; CYP93B5, AAF04115.1; CYP93B14, ACB56919.1; CYP93B2, AAD39549.1; CYP93B17, BAF49323.1; CYP93B3, BAA84071.1; CYP93B24, [AMW91728.1](https://www.ncbi.nlm.nih.gov/protein/1016106396); CYP93B23, AGF30365.1; CYP93B6, BAB59004.1; CYP93B25, AJD25217. The Genebank accession numbers for the four CYP93B like proteins in *Lonicera japonica*, *Lonicera macranthoides*, and *Ziziphora bungeana* were shown as taxa names. Different groups of CYP93Bs were highlighted with the black color of light blue, light green and light orange, respectively. The CYP93B like protein (or FNSII enzyme) acquired in this study in *Ziziphora bungeana* was highlighted with red color.
